# Association between circulating fatty acid metabolites and asthma risk: a two-sample bidirectional Mendelian randomization study

**DOI:** 10.1186/s12920-023-01545-4

**Published:** 2023-05-23

**Authors:** Tingting Huang, Yichen Long, Yang Ou, Jia Li, Yilin Huang, Jinming Gao

**Affiliations:** 1 Department of Respiratory and Critical Care Medicine, Peking Union Medical College Hospital, Chinese Academy of Medical Sciences and Peking Union Medical College, Beijing, China; 2 Department of Epidemiology, School of Public Health, Southeast University, Jiangsu Nanjing, China; 3 Center for Tumor Diagnosis and Therapy, Jinshan Hospital, Fudan University, Shanghai, China; 4 Department of Respiratory and Critical Care Medicine, Peking Union Medical College Hospital, Beijing, China

**Keywords:** Genome-wide association study, Mendelian randomization, Asthma, Fatty acids, Fatty acid desaturase gene

## Abstract

**Background:**

Fatty acids are involved in a wide range of immunological responses in humans. Supplementation of polyunsaturated fatty acids has been reported to help alleviate symptoms and airway inflammation in asthma patients, whereas the effects of fatty acids on the actual risk of asthma remain controversial. This study comprehensively investigated the causal effects of serum fatty acids on asthma risk using two-sample bidirectional Mendelian Randomization (MR) analysis.

**Methods:**

Genetic variants strongly associated with 123 circulating fatty acid metabolites were extracted as instrumental variables, and a large GWAS data of asthma was used to test effects of the metabolites on this outcome. The inverse-variance weighted method was used for primary MR analysis. The weighted median, MR-Egger regression, MR-PRESSO, and leave-one-out analyses were utilized to evaluate heterogeneity and pleiotropy. Potential confounders were adjusted by performing multivariable MR analyses. Reverse MR analysis was also conducted to estimate the causal effect of asthma on candidate fatty acid metabolites. Further, we performed colocalization analysis to examine the pleiotropy of variants within the fatty acid desaturase 1 (FADS1) locus between the significant metabolite traits and the risk of asthma. Cis-eQTL-MR and colocalization analysis were also performed to determine the association between RNA expression of *FADS1* and asthma.

**Results:**

Genetically instrumented higher average number of methylene groups was causally associated with a lower risk of asthma in primary MR analysis, while inversely, the higher ratio of bis-allylic groups to double bonds and the higher ratio of bis-allylic groups to total fatty acids, were associated with higher probabilities of asthma. Consistent results were obtained in multivariable MR when adjusted for potential confounders. However, these effects were completely eliminated after SNPs correlated with the *FADS1* gene were excluded. The reverse MR also found no causal association. The colocalization analysis suggested that the three candidate metabolite traits and asthma likely share causal variants within the *FADS1* locus. In addition, the cis-eQTL-MR and colocalization analyses demonstrated a causal association and shared causal variants between *FADS1* expression and asthma.

**Conclusions:**

Our study supports a negative association between several PUFA traits and the risk of asthma. However, this association is largely attributed to the influence of *FADS1* polymorphisms. The results of this MR study should be carefully interpreted given the pleiotropy of SNPs associated with *FADS1*.

**Supplementary Information:**

The online version contains supplementary material available at 10.1186/s12920-023-01545-4.

## Introduction

Asthma is one of the most common non-communicable respiratory diseases affecting different populations across the globe, and its prevalence continues to increase. Asthma patients often experience respiratory symptoms such as wheezing, shortness of breath, chest tightness, coughing, and different intensity of variable expiratory airflow limitation [1]. Although the current stepwise treatment helps most patients achieve symptoms control, some are still at risk of recurrence and acute exacerbation. These conditions can be life-threatening even with high doses of inhaled corticosteroids [2]. A previous study in 2008 indicated that more than 50% of asthma patients have poorly controlled symptoms [3]. Although this proportion has decreased with the improvement of compliance education and the introduction of biologic therapy, there are still approximately 3–10% of asthma patients diagnosed with severe asthma according to the Global Initiative for Asthma (GINA) 2021 [1]. This seriously affects the living quality of those patients and causes a huge economic burden on individuals as well as society. Improving overall control levels of asthma is of great significance for prognosis and reducing medical costs. Further evidence regarding biomarkers linked to asthma pathogenesis and the development of novel drug targets are required.

As essential components of the human body, fatty acids have been recognized to be involved in a wide range of human immunological responses for nearly 30 years [4]. Polyunsaturated fatty acids (PUFAs), especially omega-3 and omega-6 PUFAs, have been reported to be associated with cardiovascular disease [5, 6], cholelithiasis [7], diabetes [8, 9], and many other disorders [10] in both observational studies and randomized control trials (RCTs). Dietary change is one of several environmental factors contributed to the rise of asthma incidence over the past decades, and it has been previously found that the omega-3 fatty acids eicosapentaenoic acid (EPA) and docosahexaenoic acid (DHA) are enriched in the airway mucosa and can be affected by exogenous intake [11]. Furthermore, a recent study indicated that a member of the G protein-coupled receptor (GPCR) family, FFA4, which generally functions as a receptor for a range of circulating unsaturated free long-chain fatty acids, is abundantly expressed in human bronchial epithelial cells and airway smooth muscle [12]. With its capacity of relaxing airway smooth muscle once activated, FFA4 may be a therapeutic target for asthma [13, 14]. Taken together, the role of fatty acids in asthma pathogenesis has become a promising topic of research.

Several RCTs have indicated that modification of dietary fatty acid composition by increasing omega-3 PUFAs intake and/or decreasing omega-6 PUFAs consumption has a positive effect on bronchial inflammation alleviation, such as reduction in exhaled nitric oxide, serum eosinophils, urinary leukotriene E4, and inflammatory cytokine levels [15–18]. However, clinical data evaluating the effect of fatty acids on asthma risk remains unclear. A cross-sectional study of 642 subjects conducted by Adams et al. suggested that supplementation with omega-3 PUFAs had significant correlation with reduction of bronchial hyperresponsiveness incidence, whereas n-6 PUFA levels were positively correlated with an increased risk of bronchial hyperresponsiveness due to its proinflammatory properties [19]. Controversially, an observational study carried out by Almqvist et al. presented evidence that exposure to omega-3 and omega-6 PUFAs in early life had no association with respiratory or allergic outcomes [20]. Studies on the fatty acids composition of maternal milk have also shown no straightforward evidence of association between exposure to these fatty acids during pregnancy and childhood asthma [21]. The inconclusive findings of previous studies may be due to variability in study population, sample size, measurement bias, as well as unmeasured confounding factors. Therefore, a more sensitive approach is required to fully appreciate the role of fatty acids in the development of asthma.

Mendelian randomization (MR) analysis is a genetic epidemiological study tool that has been widely used to estimate the causal association between multiple exposures and disease outcomes. MR analysis can be regarded as a “natural” randomized controlled trial that employs genetic variants (single nucleotide polymorphism, SNP) robustly associated with the exposure factor to act as proxies (instrumental variables, IVs) to assess causal associations between exposure factor and the outcome [22, 23]. Theoretically, genetic variants are randomly allocated at conception, which ensures that individuals are randomly assigned to different levels of exposure, that these levels are fixed throughout life, and are independent of other confounders, such as age, sex, environmental exposures, education status, or behavioral factors. Therefore, MR study is less likely to be affected by confounding. Simultaneously, MR analysis could avoid bias by checking and excluding pleiotropy variants, and could overcome the problem of reverse causation in traditional observation epidemiological studies [24]. This study used two-sample bidirectional and multivariable MR analysis to focus on understanding the causal effect between serum concentrations of fatty acid metabolites and the development of asthma, and to explore potential predictive biomarkers or therapeutic targets for this common disease. To our knowledge, this was the first large-scale study to evaluate the causal association of serum fatty acid metabolites with the risk of asthma.

## Materials and methods

### Study design

**Fig. 1:**
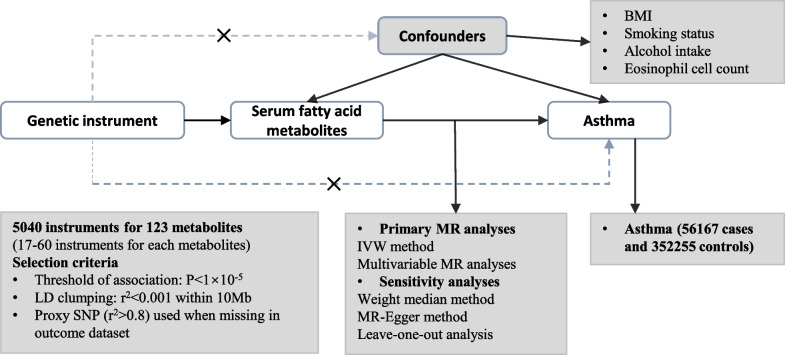
Study overview and MR model. Three principal assumptions of valid genetic instrument for MR analysis. The dotted line and the “ × ” represent the variables are invalid if they are associated with the outcome or potential confounders. IVs, instrumental variables; IVW, inverse variance weighted

The overall study design is shown in Fig. 1. The key component of our study was to examine the causal role of genetically predicted serum concentrations of fatty acid metabolites in asthma susceptibility. Our MR analysis variables were constructed to meet the following assumptions for valid genetic instrument variables [22]: (i) SNPs are robustly associated with the exposure, (ii) SNPs are independent of any confounders potentially associated with the exposure and outcome, and (iii) SNPs are independent of the outcome when the exposure and confounders exist, which associated with the outcome only by their effects on the exposure.

### Data sources

Details of the contributing GWAS consortiums are listed in Table S1 (Additional file 1). All GWAS summary data analyzed in this study were extracted from the Integrative Epidemiology Unit (IEU) database (https://gwas.mrcieu.ac.uk/), which is a public database containing 244,880,022,120 genetic associations from 42,334 GWAS summary datasets, all of the participants involved in the database have obtained ethical approval and provided written informed consent. Genetic instruments for circulating fatty acid metabolites were extracted to act as unbiased markers of exposure [25], which includes large-scale genome-wide association study (GWAS) summary data for 123 circulating metabolic traits of 24,925 individuals from the Finnish population [26]. Blood lipid and metabolite concentrations were quantified using high-throughput nuclear magnetic resonance spectroscopy metabolomics. The asthma dataset included GWAS summary statistics of 408,422 participants of White British ancestry (56,167 cases and 352,255 controls) were used to test effects of the metabolites on this outcome [27]. Asthma cases included patients with a diagnosis from hospital record (ICD-10: G47.3, ICD-9: 3472A) or primary care medical record as well as those with self-reported asthma [27]. There were no individuals who overlap between the asthma and metabolite datasets. We also extracted GWAS datasets of the shared cause of asthma and metabolites for confounder adjustment, including GWAS datasets of BMI and smoking status from a cohort of 1.2 million European individuals [28], GWAS datasets of alcohol intake from UK biobank, and eosinophil counts from a GWAS of human blood cell trait variations with 29 blood cell phenotypes in 563,085 European-ancestry participants [29].

### Selection of genetic instruments

All relevant SNPs selected as genetic instruments satisfied a relatively relaxed threshold of P < 1 × 10^–5^, which was adopted when only a few significant SNPs (P < 5 × 10^–8^) were available in the MR analysis [30–32]. Linkage disequilibrium (LD) clumping was performed on the candidate set of instrumental SNPs (SNPs with more liberal GWAS p-value of less than 1 $\times$ 10^–5^) to identify independent SNPs (r^2^ < 0.001 within 10 Mb) using data from the 1000 Genomes Project as reference panel. We then harmonized the exposure and outcome datasets to obtain genetic instrument effects on asthma and to remove palindromic SNPs. Proxy SNPs (LD at r^2^ > 0.8) were used when SNPs available for predicting a specific fatty acid metabolite in the outcome GWAS were absent. The proportion of variance (R^2^) explained by the SNPs and the F statistic were used to verify the strength of instruments for exposures [33]. Generally, SNPs with F statistic > 10 are considered sufficiently strong instruments for MR analysis [34].

### Statistical analysis

All MR analyses were performed using the TwoSampleMR package in R software 4.2.1 [35]. Primary MR analysis was performed using the inverse-variance weighted method (IVW) to provide overall causal estimate of the impact of genetically predicted metabolite concentrations on asthma, the effect size was reported as the effect of a one-standard-deviation (1-SD) change in the metabolites. However, the IVW method can provide valid estimation only in the absence of both heterogeneity and pleiotropy, or it might introduce bias [36]. To avoid the effect of selection bias for genetic variants with pleiotropic effects on the outcome, sensitivity analyses including the MR-Egger, weighted median, MR pleiotropy residual sum and outlier (MR-PRESSO), and leave-one-out analysis were applied to detect pleiotropy and heterogeneity, and to examine the robustness of the primary results. MR-Egger regression provided valid consistent estimates if directional pleiotropy was detected [37], while weighted median method was adopted to give a valid test when up to 50% of the instruments were valid despite the existence of pleiotropy and heterogeneity [38]. Meanwhile, MR-PRESSO analyses were conducted to check and remove pleiotropy variants [39], and leave-one-out analysis was conducted to assess the impact of each variable on the causal estimates by excluding one SNP at a time from the analysis to identify whether the results were driven by a single SNP.

We performed a replication MR analysis by using two other metabolites GWAS from the IEU database as the exposure datasets to cross-validate the reliability of our results, which consists of GWAS summary data for more than 400 circulating metabolites of 7824 and 114,999 individuals from the European population, respectively [40, 41]. In addition, reverse MR analysis was conducted to estimate the causal effect of asthma on candidate fatty acid metabolites.

To examine the pleiotropy of variants within *fatty acid desaturase 1 (FADS1)* locus between the significant metabolite traits and the risk of asthma, we selected SNPs within ± 100 kb of *FADS*’s genome position [43] and conducted colocalization analysis. The coloc R package with default priors was used to perform the colocalization analysis, and the significant colocalization (posterior probability) was set at PP.H4 > 0.95, which meant the probability should be more than 95% to consider that the gene was strongly colocalized with asthma and the significant metabolite traits.

We also performed the cis-eQTL(expression quantitative trait loci)-MR and colocalization analysis to further determine the association between the RNA expression of *FADS1* and asthma. We obtained fully statistically significant cis-eQTL (FDR < 0.05, ± 1 Mb from each probe) from the eQTLGen Consortium and eQTL meta-analysis of the *FADS1* gene from the peripheral blood of 31,684 individuals [42], and selected cis-eQTL within ± 100 kb from the gene’s genome position [43].

Finally, potential confounders which could simultaneously affect the incidence of asthma and fatty acid metabolite concentrations, including BMI, smoking status, alcohol intake [44], and the eosinophil counts as a risk factor of asthma which could be affected by fatty acid metabolites [45], were statistically adjusted using regression-based multivariable MR approach to obtain direct effects of metabolites on asthma. Statistical significance was defined as P < 0.05. The FDR correction (Q-value) was performed to correct the P value of multiple tests using the Benjamini–Hochberg method.

### Ethics

Our analyses used freely accessible data from published studies and the GWAS summary database. No original data were collected for the present study and ethics committee approval was not required. Data acquisition was approved by the individual institutional ethics review committees and written informed consent had been provided by all participants.

## Results

A total of 5040 instruments for 123 metabolites were used for the primary MR analysis. After checking for validity, we extracted 17–60 SNPs responsible for an average R^2^ of 12.53% (min: 4.10%, max: 20.65%), and the average F statistic was 67.33 (min: 37.85, max: 84.25), suggesting that all SNPs were sufficiently strong instruments (F statistic > 10) for MR analysis (Additional file 1: Table S2). Among all instruments included in preliminary analysis, only three genetically determined level of fatty acid metabolite traits (the average number of methylene groups in a fatty acid chain, the ratio of bis-allylic groups to double bonds, and the ratio of bis-allylic groups to total fatty acids) were causally associated with the risk of asthma (FDR < 0.05; Table 1; Additional file 1: Figure S1). As for other metabolites, including the genetically predicted serum concentration of omega-3 fatty acids, omega-6 fatty acids, linoleic acid, apolipoprotein A/B, mono-unsaturated fatty acids, saturated fatty acids, triglycerides, phosphor-glycerides, and free cholesterol…, no significant associations were found with the occurrence of asthma in primary analysis (Additional file 1: Table S2).

**Table 1:** Mendelian randomization results and heterogeneity analysis for effect of genetically predicted fatty acid metabolites on asthma

Method	No. of SNPs	MR analysis		Heterogeneity test			MR-Egger intercept P
		OR (95% CI)	P	Cochran’s Q	I^2^	P	
*Ratio of bis-allylic groups to double bonds*							
IVW	35	1.05 (1.03–1.08)	2.09 × 10^–4^	47.24	28.03%	0.07	
Weighted Median	35	1.10 (1.06–1.14)	1.67 × 10^–7^	–	–	–	
MR-Egger	35	1.09 (1.04–1.14)	5.75 × 10^–4^	42.81	22.91%	0.12	0.07
*Ratio of bis-allylic groups to total fatty acids*							
IVW	34	1.06 (1.03–1.10)	3.3 × 10^–4^	71.54	53.87%	8.11 × 10^–5^	
Weighted Median	34	1.12 (1.07–1.16)	1.52 × 10^–8^				
MR-Egger	34	1.07 (1.02–1.13)	1.6 × 10^–2^	71.28	55.11%	1.15 × 10^–4^	0.73
*Average number of methylene groups in a fatty acid*							
IVW	30	0.92 (0.88–0.96)	4.69 × 10^–5^	47.46	38.90%	1.67 × 10^–2^	
Weighted Median	30	0.93 (0.87–0.98)	1.12 × 10^–2^				
MR-Egger	30	0.86 (0.81–0.93)	2.69 × 10^–4^	41.36	32.30%	4.97 × 10^–2^	0.05

Results from primary two sample MR analysis, estimated associations reported as odds ratio (OR) of outcome per unit increase in log odds of the quantification of specific traits; No. of SNPs refers to the number of valid instrumental variables for each exposure included in final MR analysis

*MR* Mendelian randomization

### Effect of fatty acid metabolites on asthma

**Fig. 2:**
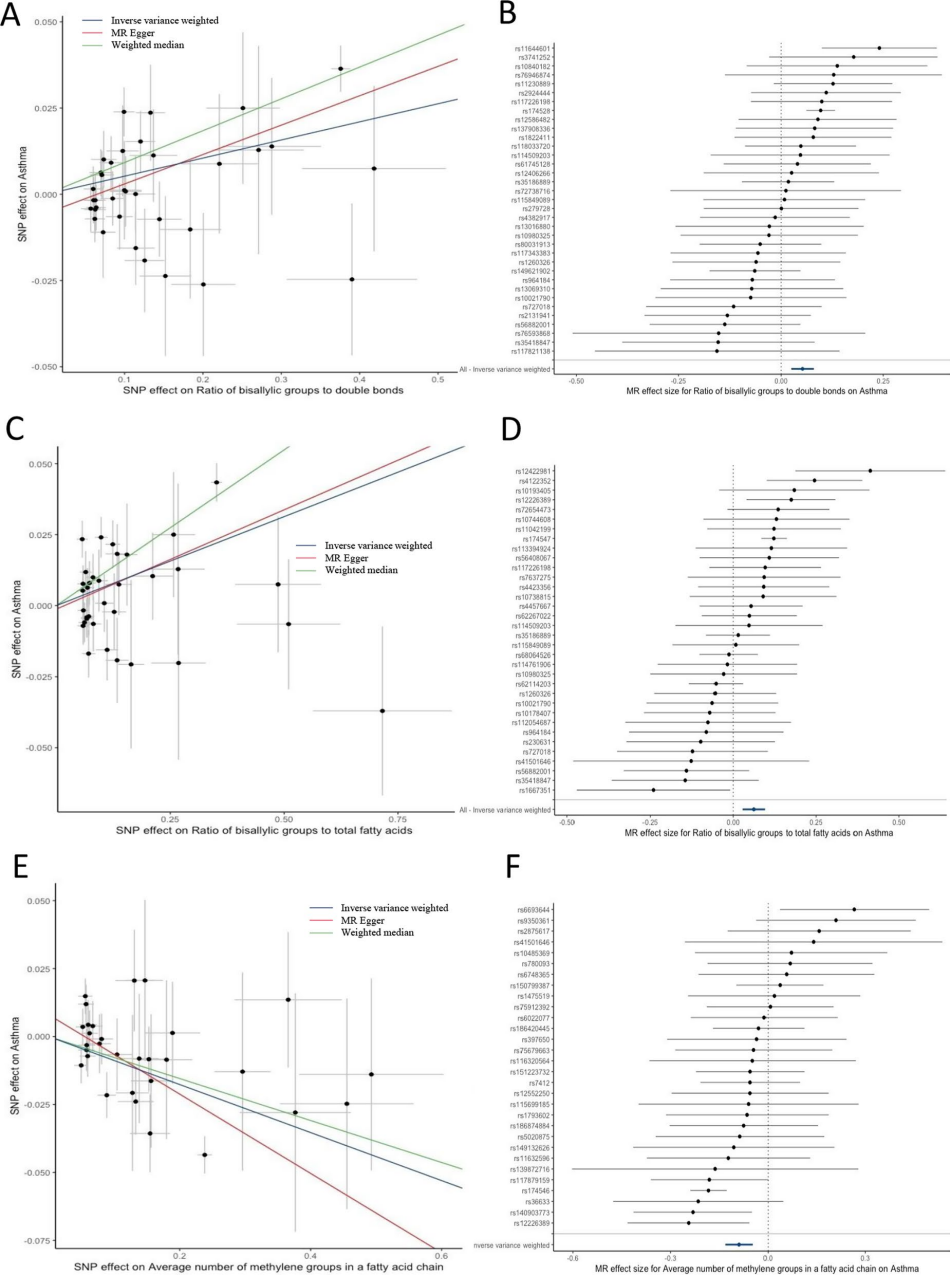
Scatter plot and forest plot of different metabolite traits’ effect on asthma. Scatter plots and forest plots of MR-derived associations of genetically predicted levels of the ratio of bis-allylic groups to double bonds **A**, **B**, the ratio of bis-allylic groups to total fatty acids **C**, **D** and the average number of methylene groups in fatty acid chain **E**–**F** versus asthma, calculated using the inverse variance weighted (IVW), weighted median, and MR-Egger methods

There were 36, 35, and 31 SNPs individually associated with the ratio of bis-allylic groups to double bonds, the ratio of bis-allylic groups to total fatty acids, and the average number of methylene groups in a fatty acid chain selected as genetic instruments (Additional file 1: Table S3-S5). These SNPs explained 0.71%, 0.69%, and 0.62% of the total variation, respectively. After one palindromic SNP with intermediate allele frequency was excluded, we observed that each 1- SD increase in the ratio of bis-allylic groups to double bonds resulted in 5% higher odds of asthma (IVW; odds ratio [OR]: 1.05, 95% CI 1.03–1.08; P = 2.09 × 10^–4^; Table 1; Figs. 2A, B). Further, each 1-SD increase in the ratio of bis-allylic groups to total fatty acids was accompanied by 6% higher odds of asthma (IVW; OR: 1.06; 95% CI 1.03–1.10; P = 3.27 × 10^–4^; Table 1; Figs. 2C, D). In contrast, a 1-SD increase in the average number of methylene groups in a fatty acid chain was associated with approximately 10% lower odds of asthma among all participants (IVW; OR: 0.92; 95% CI 0.88–0.96; P = 4.69 × 10^–5^; Table 1; Figs. 2E, F).

**Fig. 3:**
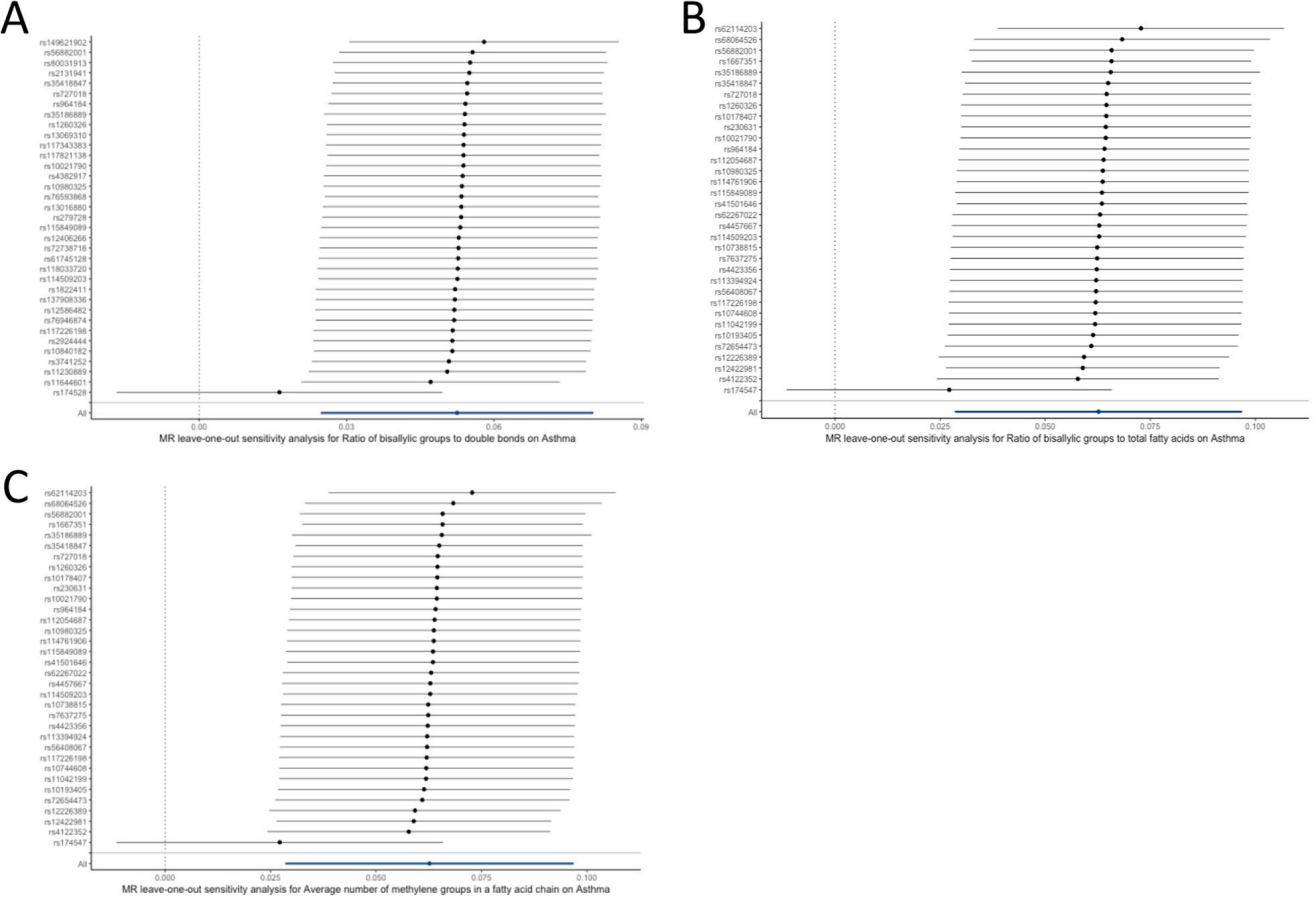
Leave-one-out analysis of the Mendelian randomization (MR) outcome. Leave-one-out analysis indicates fluctuant MR associations of the ratio of bis-allylic groups to double bonds versus asthma **A**, the ratio of bis-allylic groups to total fatty acids versus asthma (**B**) and the average number of methylene groups in fatty acid chain versus asthma (**C**)

**Fig. 4:**
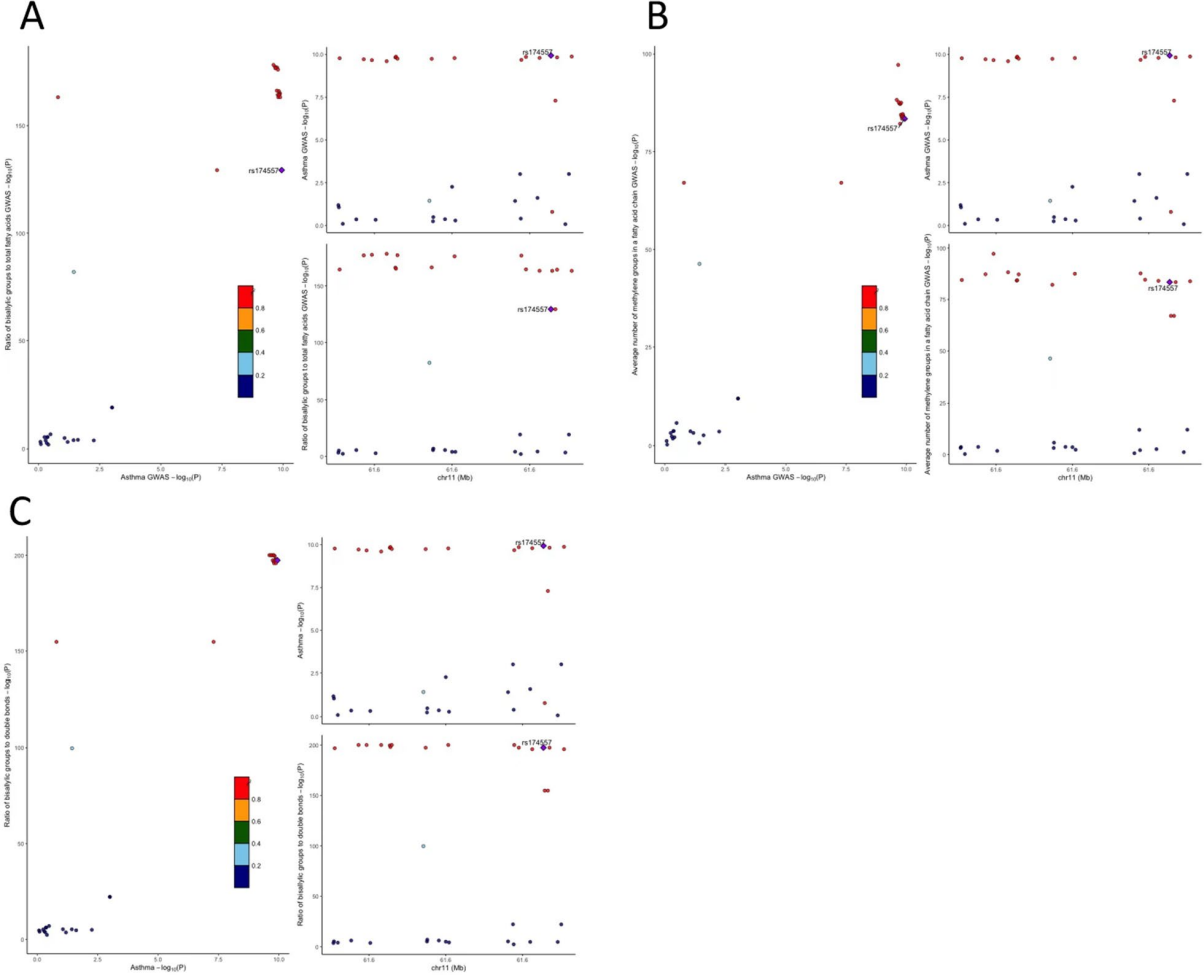
Regional Manhattan plot of associations of SNPs within FADS1 locus. Regional Manhattan plot of SNPs within *FADS1* locus and the associations between (**A**). Ratio of bis-allylic groups to total fatty acids, (**B**). Average number of methylene groups in a fatty acid chain, (**C**). Ratio of bis-allylic groups to double bonds and the risk of asthma. The significant posterior probability was set at PP.H4 > 0.95

In addition to the primary MR analysis, the MR-Egger and weighted median modes also supported a consistent causal association between the ratio of bis-allylic groups to double bonds and asthma (MR-Egger OR: 1.09; 95% CI 1.04–1.14; P = 5.75 × 10^–4^; weighted median OR: 1.10; 95% CI 1.06–1.14; P = 1.69 × 10^–7^; Table 1; Fig. 2A, B). No significant heterogeneity was observed according to IVW and MR-Egger analyses (Table 1), and the symmetry of the funnel plot was consistent with the results of the heterogeneity analyses (Additional file 1: Figure S2). Moreover, horizontal pleiotropies were not found in the MR-Egger intercept test (P_intercept_ = 0.073). Similar results were observed for the causal association of the ratio of bis-allylic groups to total fatty acids (Table 1, Fig. 2C, D; Additional file 1: Figure S3), and of the average number of methylene groups in a fatty acid chain (Table 1, Fig. 2E, F; Additional file 1: Figure S4) on asthma, when the MR-Egger and weighted median modes were used for sensitivity analyses. Unexpectedly, in the leave-one-out analysis, we found that the effects of the three fatty acid metabolite traits on asthma risk were fully attenuated when SNPs related to *fatty acid desaturase 1* (*FADS1*) were excluded (Fig. 3A–C), indicating that the main IVW results were strongly driven by the polymorphisms (rs174528, rs174546, rs174547) correlated with *FADS1*. Based on this finding, we calculated that the rs174528 and rs174546 explained the maximum R^2^ of instrument variables of the ratio of bis-allylic groups to double bonds and the average number of methylene groups in a fatty acid chain, with a proportion of 4.48% and 5.44%, respectively; while rs174547 explained 4.2% of the R^2^ of the ratio of bis-allylic groups to total fatty acids. In addition, the result of the colocalization analysis suggested that the three significant metabolite traits and asthma likely share causal variants within the FADS1 locus ('Ratio of bis-allylic groups to double bonds’: PP.H4 = 0.983; 'Ratio of bis-allylic groups to total fatty acids’: PP.H4 = 0.980; 'Average number of methylene groups in a fatty acid chain’: PP.H4 = 0.981, Fig. 4A–C).

### Replication analysis

Primary IVW analysis results of the replication analysis showed that the arachidonate (20:4n6), the ratio of polyunsaturated fatty acids to total fatty acids, the ratio of docosahexaenoic acid to total fatty acids, and the degree of unsaturation were negatively associated with the risk of asthma. The estimates remained stable in the MR Egger and Weighted median analyses. However, consistent with the main MR analysis, leave-one-out analysis revealed that the main causal associations were directly driven by SNPs related to the *FADS1* gene; removal of those SNPs resulted in non-significant results (Additional file 1: Table S6, Figure S5 A-C, Figure S6 A-G).

### Association between expression of FADS1 mRNA and asthma

**Fig. 5:**
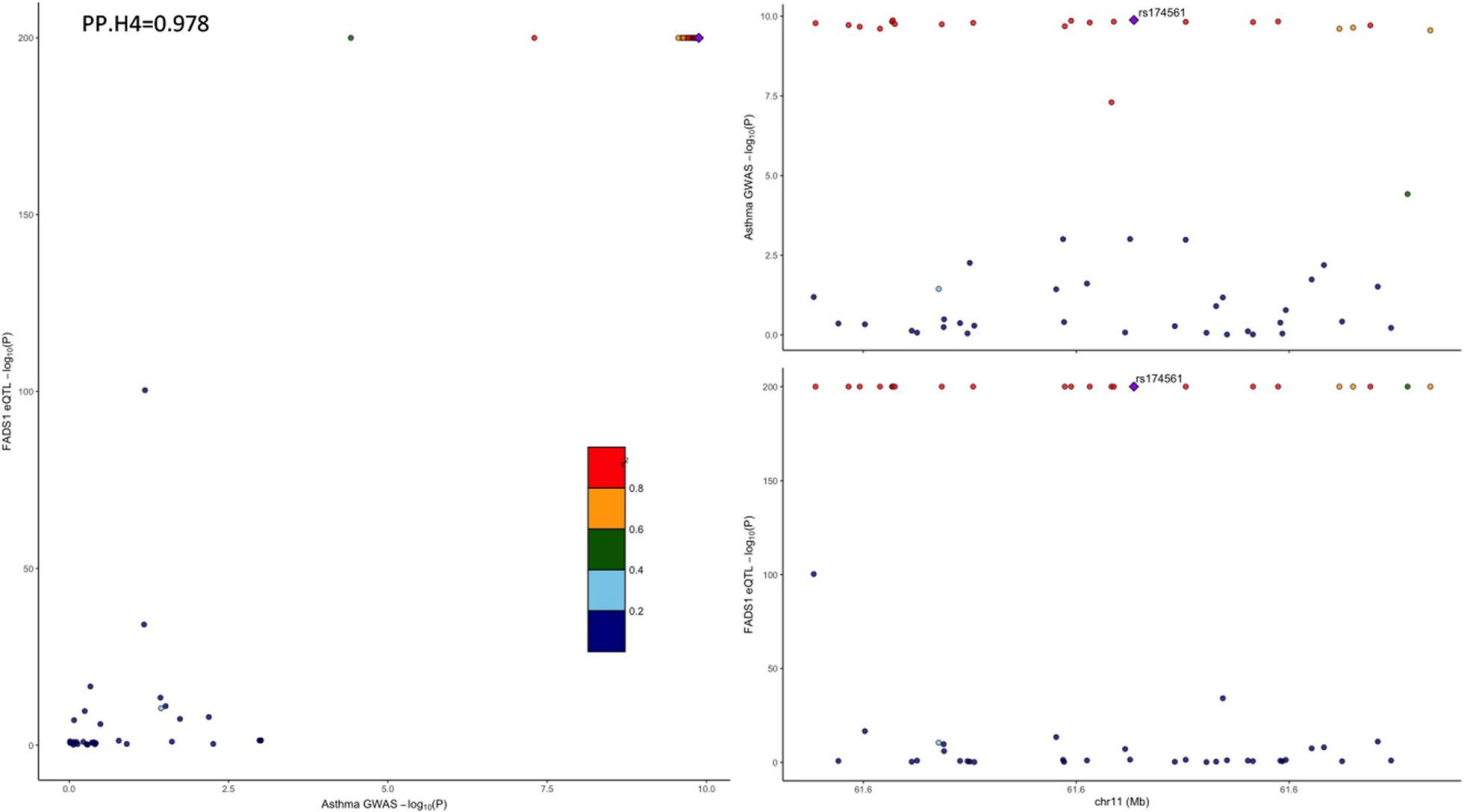
Regional Manhattan plot of associations of SNPs with FADS1. Regional Manhattan plot of SNPs within *FADS1* locus and the associations between *FADS1* mRNA and the risk of asthma. The significant posterior probability was set at PP.H4 > 0.95

The link between the *FADS1* gene and PUFA biosynthesis has been well established by previous studies [46, 47]. Based on the MR findings stated above, we additionally conducted the cis-eQTL-MR and colocalization analysis focused on the association between the *FADS1* gene expression and asthma. Cis-eQTL-MR found that the genetically predicted expression level of *FADS1* gene was negatively associated with the risk of asthma, with no presence of heterogeneity or pleiotropy detected by the MR-Egger intercept and the MR-PRESSO method (P_intercept_ = 0.304, P_MR-PRESSO global test_ = 0.36; Additional file 1: Table S7, Figure S7 A-B). The leave-out-out analysis demonstrated that the causal effect was not affected by any single SNP (Figure S7 C). In addition, the result of the colocalization analysis suggested that *FADS1* and asthma likely share causal variants within the *FADS1* locus (PP.H4 = 0.978, Fig. 5).

### Reverse MR analysis

Reverse MR analysis was further performed to evaluate the effect of asthma on the three candidate fatty acid metabolites of the forward MR analysis. SNPs met a threshold of P<5×10^−8^ were selected as genetic instruments strongly associated with asthma, and used for LD clumping. Nevertheless, lack of association was obtained between genetic liability to asthma with change of the candidate metabolite concentrations in the IVW analysis, as well as in the weighted median and MR-Egger analyses (Table 2).

**Table 2:** Mendelian randomization results for effect of genetic liability to asthma on fatty acid metabolites

Outcome	Exposure	Method	P value	Beta	OR
Ratio of bisallylic groups to double bonds	Asthma	MR Egger	0.45	− 0.2 (− 0.71, 0.31)	0.82 (0.49, 1.37)
		Weighted median	0.83	0.01 (− 0.07, 0.09)	1.01 (0.93, 1.09)
		Inverse variance weighted	0.38	0.09 (− 0.11, 0.28)	1.09 (0.9, 1.33)
Ratio of bisallylic groups to total fatty acids	Asthma	MR Egger	0.53	− 0.14 (− 0.57, 0.29)	0,87 (0.56, 1.34)
		Weighted median	0.49	0.03 (− 0.05, 0.12)	1.03 (0.95, 1.12)
		Inverse variance weighted	0.43	0.07 (− 0.1, 0.23)	1.07 (0.90, 1.26)
Average number of methylene groups in a fatty acid chain	Asthma	MR Egger	0.72	0.05 (− 0.23, 0.34)	1.05 (0.79, 1.4)
		Weighted median	0.79	0.01 (− 0.06, 0.08)	1.01 (0.94, 1.08)
		Inverse variance weighted	0.39	− 0.05 (− 0.15, 0.06)	0.95 (0.86, 1.06)

### Confounder adjustment

**Fig. 6:**
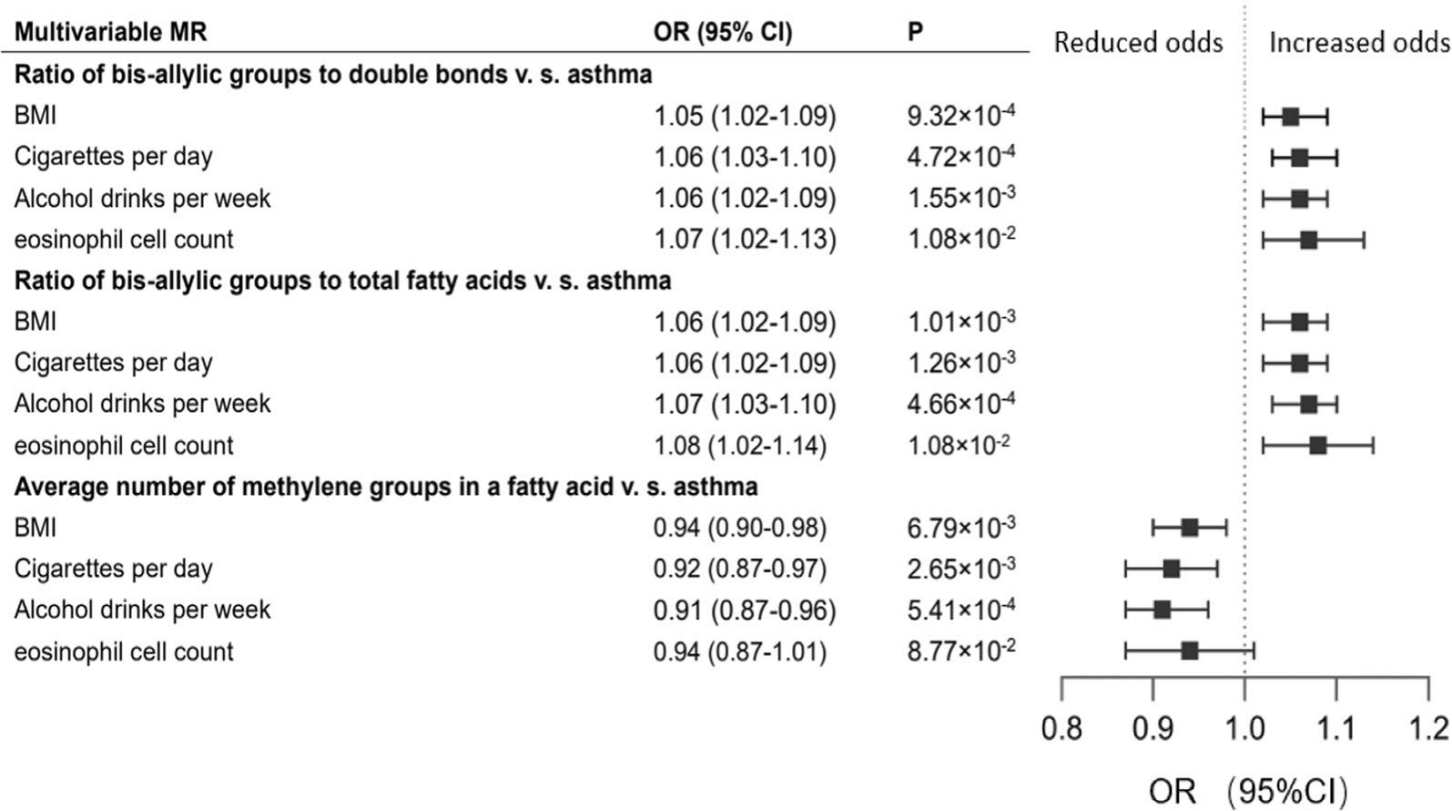
Multivariate MR adjustment for associations between causal risk fatty acids traits and asthma. Estimates reported as odds ratios (OR) of asthma per 1-SD increase in quantification of specific metabolite traits, accounting for BMI, smoking status, alcohol intake and eosinophil count

We further conducted multivariable MR analyses to adjust for confounders. All variable instruments for confounders met the relatively liberal threshold of P < 1 × 10^–5^. The causal effects of the ratio of bis-allylic groups to double bonds on asthma were broadly consistent when adjusted for BMI (OR: 1.05; 95% CI 1.02–1.09; P = 9.32 × 10^–4^, Fig. 6), smoking status (OR: 1.06; 95% CI 1.03–1.10; P = 4.72 × 10^–4^, Fig. 6), alcohol intake (OR: 1.07; 95% CI 1.03–1.10; P = 4.66 × 10^–4^, Fig. 6), and eosinophil counts (OR: 1.07; 95% CI 1.02–1.13; P = 1.08 × 10^–2^, Fig. 6). The results of the causal association between the ratio of bis-allylic groups to total fatty acids and asthma were also robust when adjusted for BMI (OR: 1.06; 95% CI 1.02–1.09; P = 1.01 × 10^–3^, Fig. 6), smoking status (OR: 1.06; 95% CI 1.02–1.09; P = 1.26 × 10^–3^, Fig. 6), alcohol intake (OR: 1.06; 95% CI 1.02–1.09; P = 1.55 × 10^–3^, Fig. 6), and eosinophil counts (OR: 1.08; 95% CI 1.02–1.14; P = 1.08 × 10^–2^, Fig. 6). Consistent results were displayed for the effects of the average number of methylene groups in a fatty acid chain on asthma after adjusting for every single confounder, except for eosinophil counts which had broader CIs (Fig. 6).

## Discussion

Previous observational and clinical studies have shown that supplementation with PUFAs, especially omega-3 fatty acids, may have a protective impact on symptom control and airway inflammation reduction in asthma. However, there is little evidence in the literature with regards to whether fatty acid composition in vivo has a causal association with asthma risk. This study aimed to comprehensively evaluate the association between fatty acids and asthma risk from the perspective of genetic epidemiology by focusing on the metabolite concentrations of fatty acids and applying the bidirectional and multivariable MR approach as a well-powered analysis tool.

Methylene groups and double bonds are different structural components of fatty acids; the number of methylene groups reflects the chain length of a fatty acid while the number of double bonds is related to the degree of unsaturation. As for the bis-allylic groups, it has been demonstrated in many studies that the bis-allylic hydrogen atoms are easily abstracted by peroxidase and participate in lipid peroxidation, and the oxidation products can induce inflammatory responses in almost all tissues, including pulmonary tissues [48, 49]. Based on the results of previous research on PUFAs and FFA4, we speculated that traits related to the degree of unsaturation and chain length of fatty acid might play a protective role in asthma risk, whereas the bis-allylic group might promote the risk of asthma.

In the primary analysis, we demonstrated that higher genetically predicted levels of the average number of methylene groups in a fatty acid chain were causally associated with a significantly lower risk of overall asthma. In contrast, traits related to the levels of bis-allylic groups in fatty acids were positively associated with the risk of asthma. These results were in line with our initial speculations, and were robustly consistent after adjusting for BMI, smoking status, alcohol intake and eosinophil counts. However, all causal associations were completely eliminated in the sensitivity analyses after excluding SNPs related to *FADS1* (rs174528, rs174546, rs174547). This suggests that all causality above was driven by the polymorphisms correlated with *FADS1* and that there is no evidence of a direct causal effect of fatty acids on the risk of asthma. The replication analysis also obtained consistent results, and the Cis-eQTL-MR demonstrated that the higher genetically predicted expression level of the *FADS1* gene was causally associated with a lower risk of asthma. Meanwhile, the reverse MR found no causal association of genetic liability to asthma with levels of the candidate metabolites.

The *fatty acid desaturase* (*FADS*) gene encodes enzymes that catalyze the endogenous conversion of upstream fatty acids into EPA and DHA, in which the *FADS1* and *FADS2* genes encode the delta-5 desaturase and delta-6 desaturase, respectively [50]. Endogenous production of long-chain PUFAs depends on the conversion efficacy of precursor fatty acids by *FADS* [51]. As for the three pivotal influential SNPs in our present study, rs174546 is a prime variant in the 3′UTR region of *FADS1* while rs174547 is located in intron 9 of *FADS1*; both have been broadly reported to be robustly associated with desaturase activity and changes in plasma fatty acid profiles [50, 52–54]. Different from these two SNPs, rs174528 is an intron SNP of the *myelin regulatory factor* (*MYRF)* gene which is located upstream of *FADS1* and defined as ‘SNP nearby the *FADS* locus’ by Borges et al. [55]. Although the role of this specific SNP in disease remains to be studied, polymorphisms of the MYRF gene were associated with serum omega-3 fatty acid concentration in a GWAS study [50]. An MR analysis by Zhao et al. [56] displayed a beneficial effect of linoleic acid on asthma, but similar to our leave-one-out analysis results, the influential SNP rs99780 was a well-established genetic polymorphism for *FADS2*, and the significance was eliminated after the removal of rs99780. An observational study analyzed the association between intake of EPA and DHA during early childhood and the incidence of new cases of asthma at 11–14 years of age. The results showed that long-chain omega-3 consumption was not associated with asthma in the whole cohort, whereas the risk was significantly lower in a subgroup of children carrying a common *FADS* variant associated with lower levels of long-chain omega-3 fatty acids in the blood [57]. This evidence strongly supports our finding that asthma risk affected by serum long-chain unsaturated fatty acid concentrations was mainly induced by variants related to *FADS* rather than exogenous PUFAs intake. During our further colocalization analysis, evidence of shared causal variants within the *FADS1* locus was found between asthma and *FADS1,* which means the locus on *FADS1* can potentially affect the occurrence of asthma by regulating the biological process of the *FADS1* gene expression.

Overall, our study demonstrated that the genetically predicted traits related to concentrations of serum long-chain unsaturated fatty acids were negatively associated with the risk of asthma, but this association was genetically instrumented by *FADS1* polymorphisms. It’s difficult to make the statement that daily supplementation of long-chain PUFAs could act as a clinical measure for asthma prevention. The results of this MR study should be carefully interpreted given the fact that *FADS1* is one of the most pleiotropic loci, despite the absence of evidence of pleiotropy using various pleiotropy-robust MR methods. Although evidence from several studies suggest that supplementation with PUFAs could help reduce airway inflammation [15–18], most did not consider the role of *FADS* as a powerful influence. In-depth and rigorous clinical research based on genotype of *FADS* is needed to better understand the mechanisms underlying the associations between fatty acids and asthma. Limitations of this study must be noted here. First and foremost, the demonstrated association of asthma with the candidate metabolite in MR is instrumented by the *FADS1* gene, which is one of the most pleiotropic loci. Although the MR statistical methods did not detect pleiotropy, just the fact that the effect disappeared after removing the *FADS1* SNPs. Second, genome-wide significant SNPs available for MR analysis were scarce. Although we adopted a relatively relaxed threshold for selecting genetic instruments, they only explained the total variation with a mean of 12.53% (min: 4.10%, max: 20.65%). In addition, peripheral blood was chosen as the sample source for the metabolomics GWAS based on convenience. However, we do not know whether fatty acid concentrations have similar roles in bronchoalveolar lavage fluid, because of reported enrichment of fatty acids in airway epithelial cells. Besides, the GWAS data included in our study was mainly from cohorts of European ancestry, and therefore unable to directly extrapolate the result to other populations. Finally, our study could not determine the relevant mechanisms underlying our results; further experimental verification is needed to explore the mechanisms of the *FADS* gene and its polymorphisms in the pathogenesis of asthma.

## Conclusion

In summary, our MR findings support a significant negative association between genetically predicted circulating levels of metabolite traits related to long-chain PUFAs and the risk of asthma. However, this association is largely attributed to the influence of polymorphisms of the *FADS1* gene. The results of this MR study should be carefully interpreted given the pleiotropy of SNPs associated with *FADS1*.

## Supplementary Information

**Additional file1**: **Table S1.** Data source of genome-wide association studies included in the Mendelian randomization analysis. **Table S2.** Summary information of the instrumental variables of metabolites used for Mendelian randomization analysis. **Table S3-S5.** Identified SNPs for each exposure (ratio of bis-allylic groups to double bonds, ratio of bis-allylic groups to total fatty acids,average number of methylene groups in a fatty acid chain.**Table S6.** Replication Mendelian randomization results for effect of genetically predicted fatty acid metabolites on asthma.**Table S7. **cis-eQTL-MR results and heterogeneity analysis for effect of genetically predicted expression level of FADS1 gene on asthma.**Figure S1-S4.** volcano plot and funnel plots for each exposure.**Figure S5-S6.** Scatter plot and forest plot for replication MR analysis using two other metabolites GWAS.**Figure S7. **Scatter plot, funnel plot and forest plot of cis-eQTL-MR for the effect of FADS1 expression on asthma.

## Data Availability

All data of the datasets analyzed during the current study are accessible from the Integrative Epidemiology Unit (IEU) database (https://gwas.mrcieu.ac.uk/), the GWAS ID for the asthma dataset used in our study was ebi-a-GCST90014325, and the GWAS ID for the datasets of the average number of methylene groups in a fatty acid chain, the ratio of bis-allylic groups to double bonds, the ratio of bis-allylic groups to total fatty acids, BMI, smoking status, alcohol intake, and eosinophil counts, were met-c-848, met-c-844, met-c-845, ukb-b-19953, ieu-b-25, ieu-b-73, and ieu-b-33, respectively. The GWAS ID for other datasets of the fatty acid metabolites extracted for the primary MR analysis were presented in Supplementary Info File 1, Table S2. GWAS ID of candidate metabolites in replication analysis were met-a-319, met-d-PUFA_pct, met-d-DHA_pct, met-d-Unsaturation for arachidonate (20:4n6), ratio of polyunsaturated fatty acids to total fatty acids, ratio of docosahexaenoic acid to total fatty acids, and the degree of unsaturation, respectively. The code used for this study is openly available on GitHub (huangtingtingwsy/Mendelian-randomization-fatty-acid-asthma (github.com)).

## Abbreviations

MR: Mendelian randomization
IVW: Inverse-variance weighted method
PUFAs: Polyunsaturated fatty acids
FADS: Fatty acid desaturase
GWAS: Genome-wide association study
RCTs: Randomized control trials
eQTL: Expression quantitative trait loci
EPA: Eicosapentaenoic acid
DHA: Docosahexaenoic acid
GPCR: G protein-coupled receptor
SNP: Nucleotide polymorphism
IVs: Variables
LD: Linkage disequilibrium
OR: Odds ratio
MR-PRESSO: MR pleiotropy residual sum and outlier
SD: Standard deviation
MYRF: Myelin regulatory factor
