# Supplementary material for: Association between circulating fatty acid metabolites and asthma risk: a two-sample bidirectional Mendelian randomization study
Source: BMC Med Genomics. 2023 May 23;16:112. doi: 10.1186/s12920-023-01545-4 (PMC10204212; doi:10.1186/s12920-023-01545-4)
Supplement: Supplementary file 1 — Additional file1: Table S1. Data source of genome-wide association studies included in the Mendelian randomization analysis. Table S2. Summary information of the instrumental variables of metabolites used for Mendelian randomization analysis. Table S3-S5. Identified SNPs for each exposure (ratio of bis-allylic groups to double bonds, ratio of bis-allylic groups to total fatty acids,average number of methylene groups in a fatty acid chain.Table S6. Replication Mendelian randomization results for effect of genetically predicted fatty acid metabolites on asthma.Table S7. cis-eQTL-MR results and heterogeneity analysis for effect of genetically predicted expression level of FADS1 gene on asthma.Figure S1-S4. volcano plot and funnel plots for each exposure.Figure S5-S6. Scatter plot and forest plot for replication MR analysis using two other metabolites GWAS.Figure S7. Scatter plot, funnel plot and forest plot of cis-eQTL-MR for the effect of FADS1 expression on asthma. [file 12920_2023_1545_MOESM1_ESM.pdf]

Association between circulating fatty acid metabolites and asthma：A two-sample Mendelian randomization analysis

Huang Tingting，Long Yichen, Li Jia，Huang Yilin，Gao Jinming

Supplementary Material

Supplementary Table

Table S1. Data source of genome-wide association studies included in the Mendelian randomization analysis

Category	Phenotype	Sample Size	Cases	Controls	Population
Exposure	Plasma fatty acid metabolites	24925	-	-	European ancestry
Outcome	Asthma	408422	56167	352255	European ancestry
Confounders	BMI	461,460	-	-	European ancestry
	Smoking status	337,334	-	-	European ancestry
	Alcohol intake	335,394	-	-	European ancestry
	Eosinophil cell count	563,085	-	-	European ancestry

Table S2. Summary information of the instrumental variables of metabolites used for Mendelian randomization analysis

GWAS ID	Metabolites	No. of SNPs	BETA	P value	SE	Q value	R^2	F statistic
met-c-958	Concentration of chylomicrons and largest VLDL particles	32	-0.014733336	0.47	0.0202643	0.951899	0.092	58.0
met-c-929	Total lipids in small VLDL	51	-0.020158431	0.23	0.0169491	0.951899	0.163	70.7
met-c-838	Acetoacetate	24	-0.002430462	0.92	0.0229501	0.9914399	0.047	37.8
met-c-853	Free cholesterol to esterified cholesterol ratio	42	-0.004406817	0.82	0.0190249	0.9914399	0.159	59.0
met-c-936	Total fatty acids	40	0.004231528	0.78	0.0154053	0.9914399	0.164	64.5
met-c-876	Free cholesterol in large HDL	46	0.016872005	0.41	0.0203757	0.951899	0.141	75.4
met-c-855	Omega-3 fatty acids	24	0.059017025	0.02	0.0259337	0.4017662	0.098	61.0
met-c-919	Phenylalanine	34	-0.027231089	0.33	0.0279657	0.951899	0.081	58.8
met-c-865	Mean diameter for HDL particles	45	0.010670357	0.52	0.0166477	0.951899	0.149	71.7
met-c-918	Phosphatidylcholine and other cholines	41	-0.022298733	0.13	0.0147715	0.951899	0.166	65.4
met-c-943	Cholesterol esters in very large HDL	33	0.014221975	0.58	0.0256861	0.951899	0.105	68.5
met-c-935	Sphingomyelins	37	-0.022530231	0.16	0.0159498	0.951899	0.119	49.1
met-c-913	Concentration of medium VLDL particles	44	0.003421244	0.85	0.0175765	0.9914399	0.142	68.9
met-c-883	Total lipids in large LDL	53	-0.001363218	0.92	0.0137516	0.9914399	0.177	76.7
met-c-866	Histidine	35	0.029988248	0.18	0.0224321	0.951899	0.112	68.9
met-c-948	Triglycerides in very large HDL	42	0.000121563	0.99	0.0175893	0.9944857	0.134	77.0

met-c-914	Phospholipids in medium VLDL	46	0.010919482	0.49	0.0159975	0.951899	0.131	68.1
met-c-851	Average number of double bonds in a fatty acid chain	32	0.057894771	0.01	0.0209281	0.1394452	0.112	62.0
met-c-945	Total lipids in very large HDL	35	0.006009479	0.72	0.0167885	0.9914399	0.103	63.4
met-c-857	Omega-7, omega-9 and saturated fatty acids	35	-0.011447293	0.53	0.0180637	0.951899	0.141	59.9
met-c-957	Total lipids in chylomicrons and largest VLDL particles	31	0.000826203	0.97	0.021701	0.9914399	0.095	64.0
met-c-885	Phospholipids in large LDL	57	-0.008458624	0.57	0.0150433	0.951899	0.176	78.0
met-c-930	Concentration of small VLDL particles	53	-0.010865714	0.5	0.0159896	0.951899	0.168	70.8
met-c-960	Triglycerides in chylomicrons and largest VLDL particles	31	0.007757005	0.72	0.0212904	0.9914399	0.091	69.9
met-c-846	3-hydroxybutyrate	25	-0.000582586	0.98	0.0258586	0.9914399	0.056	57.5
met-c-937	Total phosphoglycerides	43	-0.014761266	0.42	0.0182027	0.951899	0.174	65.8
met-c-939	Urea	17	0.011224337	0.77	0.0378042	0.9914399	0.041	47.2
met-c-902	Concentration of medium HDL particles	24	-0.040102752	0.11	0.0253273	0.951899	0.071	61.7
met-c-953	Total lipids in very small VLDL	54	-0.016915748	0.31	0.0165071	0.951899	0.197	84.3
met-c-867	Total cholesterol in IDL	52	-0.007570021	0.67	0.0176069	0.9887933	0.168	73.4
met-c-959	Phospholipids in chylomicrons and largest VLDL particles	29	-0.010236477	0.75	0.031601	0.9914399	0.088	69.0
met-c-909	Total cholesterol in medium VLDL	42	-0.000363897	0.98	0.0143765	0.9914399	0.125	69.8
met-c-922	Concentration of small HDL particles	27	-0.033938375	0.28	0.0316524	0.951899	0.079	59.0

met-c-840	Alanine	39	-0.020170836	0.33	0.0205581	0.951899	0.118	82.8
met-c-895	Total cholesterol in LDL	55	-0.009701902	0.49	0.0141099	0.951899	0.167	75.7
met-c-847	Average number of methylene groups per double bond	29	-0.047199971	0.06	0.0253253	0.8522183	0.121	60.1
met-c-863	Glycoprotein acetyls	30	-0.004885652	0.85	0.0252122	0.9914399	0.092	63.1
met-c-850	Creatinine	44	-0.009831306	0.65	0.0219323	0.9887933	0.127	82.2
met-c-955	Phospholipids in very small VLDL	51	-0.001424449	0.94	0.0182757	0.9914399	0.180	79.8
met-c-927	Total cholesterol in small VLDL	49	-0.010459465	0.58	0.0190735	0.951899	0.135	66.9
met-c-940	Valine	31	0.002907061	0.88	0.0198826	0.9914399	0.088	77.4
met-c-904	Total cholesterol in medium LDL	59	-0.007850126	0.58	0.0140412	0.951899	0.171	73.7
met-c-874	Total cholesterol in large HDL	52	0.014002107	0.43	0.0178005	0.951899	0.151	73.3
met-c-894	Lactate	23	-0.060779171	0.01	0.0236464	0.2082824	0.055	60.8
met-c-932	Triglycerides in small VLDL	43	-0.007914359	0.67	0.0183818	0.9887933	0.132	74.6
met-c-887	Cholesterol esters in large VLDL	35	-0.028608362	0.3	0.0276593	0.951899	0.112	66.5
met-c-839	Acetate	29	-0.020815955	0.47	0.0287382	0.951899	0.063	57.0
met-c-942	Total cholesterol in very large HDL	36	0.017353307	0.49	0.0253773	0.951899	0.108	72.0
met-c-861	Glycerol	27	0.027541693	0.16	0.0197602	0.951899	0.064	50.8
met-c-899	Cholesterol esters in medium HDL	28	-0.020410824	0.37	0.0229557	0.951899	0.086	60.3
met-c-941	Mean diameter for VLDL particles	39	-0.020162489	0.37	0.0223351	0.951899	0.110	59.4
met-c-888	Free cholesterol in large VLDL	33	-0.019876421	0.32	0.0200114	0.951899	0.103	71.8
met-c-912	Total lipids in medium VLDL	44	-0.001926069	0.93	0.0214368	0.9914399	0.138	67.1

met-c-843	Apolipoprotein B	55	-0.000814479	0.95	0.0140087	0.9914399	0.179	77.5
met-c-954	Concentration of very small VLDL particles	44	-0.022988238	0.18	0.0169633	0.951899	0.160	79.6
met-c-854	Description of average fatty acid chain length, not actual carbon number	33	-0.016674996	0.42	0.0204919	0.951899	0.118	51.4
met-c-916	Mono-unsaturated fatty acids	35	-0.010777711	0.51	0.0163952	0.951899	0.149	63.9
met-c-849	Citrate	28	-0.014031037	0.54	0.0228627	0.951899	0.077	68.6
met-c-856	Omega-6 fatty acids	40	-0.001744574	0.92	0.0168915	0.9914399	0.159	62.3
met-c-938	Tyrosine	31	0.000663329	0.98	0.0318413	0.9914399	0.081	68.3
met-c-944	Free cholesterol in very large HDL	38	0.006446548	0.77	0.0215982	0.9914399	0.117	75.1
met-c-950	Concentration of very large VLDL particles	37	-0.023294639	0.36	0.0256226	0.951899	0.126	69.7
met-c-917	Other polyunsaturated fatty acids than 18:2	38	0.048169315	0	0.0151041	0.0438782	0.162	67.2
met-c-907	Concentration of medium LDL particles	52	-0.001632853	0.91	0.0142182	0.9914399	0.157	67.6
met-c-956	Triglycerides in very small VLDL	54	-0.009767485	0.56	0.0165793	0.951899	0.169	69.6
met-c-870	Concentration of IDL particles	52	-0.004490425	0.8	0.0178349	0.9914399	0.181	80.1
met-c-879	Phospholipids in large HDL	41	0.025241046	0.3	0.02425	0.951899	0.140	74.3
met-c-844	Ratio of bisallylic groups to double bonds	35	0.052504286	0	0.0141608	0.0128616	0.135	58.4
met-c-841	Albumin	26	-0.006734518	0.8	0.0260046	0.9914399	0.075	56.7
met-c-925	Total lipids in small LDL	55	0.003299758	0.84	0.0160391	0.9914399	0.163	68.0

met-c-848	Average number of methylene groups in a fatty acid chain	30	-0.088330224	0	0.0216992	0.0057663	0.085	56.7
met-c-900	Free cholesterol in medium HDL	32	-0.025519946	0.26	0.0227284	0.951899	0.085	62.5
met-c-860	Glutamine	42	-0.00050685	0.98	0.0186483	0.9914399	0.120	75.8
met-c-886	Total cholesterol in large VLDL	37	-0.018087833	0.35	0.0195464	0.951899	0.103	63.9
met-c-852	22:6, docosahexaenoic acid	27	0.022958396	0.41	0.0279293	0.951899	0.104	57.7
met-c-862	Glycoproteins	39	-0.006993918	0.58	0.0126997	0.951899	0.129	67.6
met-c-931	Phospholipids in small VLDL	49	0.002479465	0.88	0.0160922	0.9914399	0.143	70.5
met-c-911	Free cholesterol in medium VLDL	44	0.008557894	0.59	0.0158041	0.951899	0.120	65.6
met-c-868	Free cholesterol in IDL	49	-0.012417323	0.47	0.0171197	0.951899	0.144	72.3
met-c-908	Phospholipids in medium LDL	52	-0.001708409	0.91	0.0149399	0.9914399	0.150	70.3
met-c-891	Phospholipids in large VLDL	34	-0.019458785	0.34	0.0202179	0.951899	0.094	63.1
met-c-946	Concentration of very large HDL particles	37	0.027379084	0.2	0.0214054	0.951899	0.118	69.4
met-c-845	Ratio of bisallylic groups to total fatty acids	34	0.062683547	0	0.0174446	0.0133871	0.140	60.9
met-c-951	Phospholipids in very large VLDL	32	-0.021650647	0.33	0.0221393	0.951899	0.096	68.1
met-c-928	Free cholesterol in small VLDL	52	-0.011526734	0.53	0.0181461	0.951899	0.166	77.7
met-c-878	Concentration of large HDL particles	44	0.033632225	0.1	0.0205032	0.951899	0.146	73.3
met-c-923	Triglycerides in small HDL	40	0.003596204	0.88	0.0235043	0.9914399	0.122	71.2
met-c-921	Total lipids in small HDL	29	-0.014960777	0.61	0.0297184	0.9692875	0.088	61.9
met-c-893	18:2, linoleic acid (LA)	34	-0.023043798	0.3	0.0223956	0.951899	0.135	61.9

met-c-877	Total lipids in large HDL	42	0.03247527	0.13	0.021218	0.951899	0.144	75.4
met-c-871	Phospholipids in IDL	51	-0.011695796	0.44	0.0151136	0.951899	0.161	77.9
met-c-905	Cholesterol esters in medium LDL	55	-0.004937097	0.72	0.0138641	0.9914399	0.162	67.3
met-c-949	Total lipids in very large VLDL	36	-0.00725201	0.84	0.0353947	0.9914399	0.110	66.1
met-c-920	Pyruvate	35	-0.031359824	0.17	0.022815	0.951899	0.072	54.5
met-c-915	Triglycerides in medium VLDL	35	-0.001696088	0.95	0.0254665	0.9914399	0.106	67.8
met-c-869	Total lipids in IDL	51	-0.008218786	0.64	0.0176404	0.9859715	0.176	78.8
met-c-884	Concentration of large LDL particles	57	0.000301162	0.98	0.0134081	0.9914399	0.185	75.3
met-c-896	Mean diameter for LDL particles	35	0.02725362	0.32	0.0275131	0.951899	0.108	64.6
met-c-934	Serum total triglycerides	44	-0.018404503	0.3	0.0175849	0.951899	0.138	74.8
met-c-875	Cholesterol esters in large HDL	43	0.020448142	0.29	0.0191615	0.951899	0.144	73.6
met-c-897	Leucine	29	0.018878867	0.42	0.0233159	0.951899	0.078	70.1
met-c-858	Free cholesterol	50	-0.010889361	0.4	0.0128516	0.951899	0.182	59.9
met-c-901	Total lipids in medium HDL	28	-0.025337677	0.32	0.0255919	0.951899	0.077	57.3
met-c-926	Concentration of small LDL particles	51	-0.004357887	0.79	0.0167667	0.9914399	0.143	62.8
met-c-890	Concentration of large VLDL particles	36	0.008760909	0.71	0.0233659	0.9914399	0.102	58.4
met-c-864	Total cholesterol in HDL	38	-0.006909106	0.76	0.0229593	0.9914399	0.119	76.1
met-c-842	Apolipoprotein A-I	40	-0.022324102	0.41	0.0271205	0.951899	0.116	65.8
met-c-952	Triglycerides in very large VLDL	34	-0.033379775	0.28	0.0307226	0.951899	0.109	75.2
met-c-872	Triglycerides in IDL	60	-0.016453192	0.41	0.0198339	0.951899	0.207	82.0
met-c-947	Phospholipids in very large HDL	35	0.008302405	0.64	0.0176323	0.9859715	0.115	71.7

met-c-924	Total cholesterol in small LDL	50	-0.005303292	0.73	0.0155781	0.9914399	0.140	67.2
met-c-933	Serum total cholesterol	53	-0.012770642	0.37	0.0143391	0.951899	0.146	67.9
met-c-892	Triglycerides in large VLDL	42	-0.02073559	0.37	0.023188	0.951899	0.114	64.8
met-c-880	Total cholesterol in large LDL	51	-0.009942041	0.48	0.0140529	0.951899	0.151	72.3
met-c-889	Total lipids in large VLDL	41	-0.019144252	0.3	0.0185836	0.951899	0.112	57.9
met-c-873	Isoleucine	28	0.043935386	0.06	0.0230791	0.8522183	0.075	66.8
met-c-882	Free cholesterol in large LDL	52	-0.014610725	0.26	0.0130078	0.951899	0.153	71.8
met-c-903	Phospholipids in medium HDL	37	-0.020609327	0.38	0.0232819	0.951899	0.100	64.6
met-c-881	Cholesterol esters in large VLDL	49	-0.001262734	0.93	0.0142087	0.9914399	0.155	70.7
met-c-906	Total lipids in medium LDL	51	-0.007382898	0.61	0.0144903	0.9692875	0.156	68.1
met-c-898	Total cholesterol in medium HDL	33	0.000833041	0.97	0.0234189	0.9914399	0.088	63.1
met-c-859	Glucose	37	-0.007516741	0.73	0.0217499	0.9914399	0.088	64.4
met-c-910	Cholesterol esters in medium VLDL	49	-0.017000654	0.32	0.0170697	0.951899	0.153	68.0

Table S3. Genetic instruments (n=36) for the ratio of bis-allylic groups to double bonds and associations with asthma

**SNP ID**	**proxy SNP**	**r2 for proxy**	**Effect allele (alternative)**	**β (Standard Error) for ratio of bis-allylic groups to double bonds**	**β (Standard Error) for asthma**
rs10021790	-	-	C(T)	-0.057 (0.013)	0.004(0.007)
rs10840182	-	-	T(C)	0.073 (0.016)	0.010(0.008)
rs10980325	-	-	C(A)	0.060 (0.013)	-0.002(0.007)
rs11230889	-	-	C(A)	-0.120 (0.019)	-0.015(0.009)

rs114509203	-	-	A(G)	-0.271 (0.058)	-0.013(0.030)
rs115849089	-	-	A(G)	0.101 (0.020)	0.001(0.010)
rs11644601	-	-	C(T)	0.099(0.014)	0.024(0.007)
rs117226198	-	-	T(C)	-0.251 (0.047)	-0.025(0.022)
rs117343383	-	-	T(A)	-0.184 (0.040)	0.010(0.020)
rs117821138	-	-	T(C)	0.152 (0.033)	-0.024(0.023)
rs118033720	-	-	A(G)	-0.288 (0.063)	-0.014(0.020)
rs12406266	-	-	A(G)	-0.060 (0.013)	-0.002(0.007)
rs12586482	-	-	G(A)	-0.070 (0.015)	-0.006(0.007)
rs1260326	-	-	C(T)	0.064 (0.013)	-0.004(0.007)
rs13016880	-	-	T(C)	-0.063 (0.014)	0.002(0.007)
rs13069310	-	-	G(C)	-0.063 (0.014)	0.004(0.007)
rs137908336	-	-	A(C)	0.137 (0.031)	0.011(0.013)
rs149621902	-	-	A(G)	0.390 (0.083)	-0.025(0.022)
rs174528	-	-	C(T)	-0.376 (0.012)	-0.036(0.007)
rs1822411	-	-	T(C)	0.071 (0.015)	0.006(0.007)
rs2131941	-	-	A(C)	-0.201 (0.041)	0.026(0.021)
rs279728	-	-	T(C)	0.114 (0.025)	0.000(0.011)
rs2924444	-	-	A(G)	-0.083 (0.015)	-0.009(0.008)
rs35186889	-	-	C(T)	0.418 (0.092)	0.007(0.024)
rs35418847	-	-	T(C)	-0.126 (0.025)	0.019(0.015)
rs3741252	-	-	T(C)	0.133 (0.019)	0.024(0.014)
rs4382917	-	-	A(G)	0.085 (0.016)	-0.001(0.008)
rs56882001	-	-	A(G)	0.114 (0.024)	-0.016(0.011)
rs61745128	-	-	A(G)	0.221 (0.049)	0.009(0.020)

rs727018	-	-	T(C)	0.062 (0.013)	-0.007(0.007)
rs72738716	-	-	G(C)	-0.100 (0.022)	-0.001(0.014)
rs76593868	-	-	G(A)	-0.073 (0.016)	0.011(0.013)
rs76946874	-	-	C(T)	-0.098(0.021)	-0.013(0.013)
rs7911761	-	-	G(C)	-0.063 (0.013)	-0.025(0.007)
rs80031913	-	-	A(G)	-0.144 (0.028)	0.007(0.011)
rs964184	-	-	C(G)	0.094 (0.017)	-0.006(0.010)

Note. All SNPs with P < 1×10^{-5} clumped at $r^2 < .001$ with 10Mb.

Table S4. Genetic instruments (n=35) for the ratio of bis-allylic groups to total fatty acids and associations with asthma

**SNP ID**	**proxy SNP**	**r2 for proxy**	**Effect allele (alternative)**	**β (Standard Error) for ratio of bis-allylic groups to total fatty acids**	**β (Standard Error) for asthma**
rs10021790	-	-	C(T)	-0.067(0.013)	0.004(0.007)
rs10178407	-	-	T(C)	-0.066(0.013)	0.005(0.007)
rs10193405	-	-	A(G)	-0.064(0.013)	-0.012(0.007)
rs10738815	-	-	T(G)	-0.058(0.013)	-0.005(0.007)
rs10744608	-	-	A(G)	0.058(0.013)	0.008(0.007)
rs10980325	-	-	C(A)	0.059(0.013)	-0.002(0.007)
rs11042199	-	-	T(C)	0.080(0.017)	0.010(0.008)
rs112054687	-	-	G(A)	0.268(0.060)	-0.020(0.034)
rs113394924	-	-	G(T)	0.155(0.023)	0.018(0.018)
rs114509203	-	-	A(G)	-0.267(0.059)	-0.013(0.030)

rs114761906	-	-	A(G)	-0.127(0.027)	0.002(0.014)
rs115849089	-	-	A(G)	0.105(0.020)	0.001(0.010)
rs117226198	-	-	T(C)	-0.257(0.048)	-0.025(0.022)
rs12226389	-	-	C(T)	-0.124(0.016)	-0.022(0.008)
rs12422981	-	-	C(T)	-0.057(0.013)	-0.023(0.007)
rs1260326	-	-	C(T)	0.071(0.013)	-0.004(0.007)
rs1667351	-	-	T(A)	0.070(0.015)	-0.017(0.008)
rs174547	-	-	C(T)	-0.352(0.012)	-0.043(0.007)
rs230631	-	-	G(T)	-0.061(0.014)	0.006(0.007)
rs35186889	-	-	C(T)	0.487(0.095)	0.007(0.024)
rs35418847	-	-	T(C)	-0.133(0.026)	0.019(0.015)
rs4122352	-	-	A(G)	0.098(0.014)	0.024(0.007)
rs41501646	-	-	G(A)	0.164(0.029)	-0.021(0.030)
rs4423356	-	-	T(C)	-0.068(0.015)	-0.006(0.007)
rs4457667	-	-	T(C)	-0.137(0.029)	-0.007(0.011)
rs56408067	-	-	A(G)	-0.072(0.015)	-0.008(0.008)
rs56882001	-	-	A(G)	0.111(0.024)	-0.016(0.011)
rs62114203	-	-	T(A)	-0.716(0.153)	0.037(0.030)
rs62267022	-	-	C(G)	0.211(0.046)	0.010(0.015)
rs68064526	-	-	A(G)	-0.510(0.114)	0.007(0.023)
rs72654473	-	-	A(C)	-0.133(0.026)	-0.018(0.010)
rs727018	-	-	T(C)	0.059(0.013)	-0.007(0.007)
rs7637275	-	-	T(C)	-0.093(0.021)	-0.009(0.011)
rs7911761	-	-	G(C)	-0.059(0.013)	-0.025(0.007)
rs964184	-	-	C(G)	0.081(0.018)	-0.006(0.010)

Note. All SNPs with $P < 1\times10^{-5}$ clumped at $r^2 < .001$ with 10Mb.

Table S5. Genetic instruments (n=31) for average number of methylene groups in a fatty acid chain and associations with asthma

SNP ID	proxy SNP	r2 for proxy	Effect allele (alternative)	β (Standard Error) for average number of methylene groups in a fatty acid chain	β (Standard Error) for asthma
rs10485369	-	-	C(T)	0.060(0.013)	0.004(0.009)
rs115699185	-	-	A(G)	-0.138(0.030)	0.008(0.024)
rs116320564	-	-	A(G)	-0.180(0.040)	0.009(0.029)
rs11632596	-	-	A(T)	-0.059(0.012)	0.007(0.008)
rs117879159	-	-	A(C)	0.133(0.027)	-0.024(0.012)
rs12226389	-	-	C(T)	0.089(0.014)	-0.022(0.008)
rs12552250	-	-	A(G)	0.058(0.013)	-0.003(0.007)
rs139872716	-	-	A(G)	-0.128(0.026)	0.021(0.029)
rs140903773	-	-	G(A)	-0.155(0.031)	0.036(0.014)
rs1475519	-	-	A(G)	0.062(0.013)	0.001(0.008)
rs149132626	-	-	T(G)	-0.156(0.031)	0.016(0.025)
rs150799387	-	-	A(G)	0.366(0.082)	0.014(0.025)
rs151223732	-	-	G(T)	-0.455(0.102)	0.025(0.039)
rs174546	-	-	T(C)	0.238(0.011)	-0.044(0.007)
rs1793602	-	-	C(A)	0.104(0.023)	-0.007(0.0130
rs186420445	-	-	A(G)	-0.493(0.111)	0.014(0.035)
rs186874884	-	-	G(A)	-0.377(0.085)	0.028(0.044)

rs2875617	-	-	A(C)	0.131(0.029)	0.021(0.019)
rs36633	-	-	C(T)	-0.049(0.011)	0.011(0.007)
rs395297	-	-	T(A)	-0.050(0.011)	-0.001(0.007)
rs397650	-	-	G(A)	-0.077(0.018)	0.003(0.011)
rs41501646	-	-	G(A)	-0.147(0.028)	-0.021(0.030)
rs5020875	-	-	T(A)	0.058(0.013)	-0.005(0.008)
rs6022077	-	-	T(C)	0.081(0.018)	-0.001(0.009)
rs6693644	-	-	G(A)	0.056(0.011)	0.015(0.007)
rs6748365	-	-	C(T)	-0.067(0.015)	-0.004(0.009)
rs7412	-	-	T(C)	0.153(0.025)	-0.008(0.012)
rs75679663	-	-	A(C)	-0.296(0.043)	0.013(0.037)
rs75912392	-	-	T(C)	0.189(0.042)	0.001(0.019)
rs780093	-	-	C(T)	-0.052(0.011)	-0.004(0.007)
rs9350361	-	-	A(G)	-0.057(0.012)	-0.012(0.007)

Note. All SNPs with $P < 1\times10^{-5}$ clumped at $r^2 < .001$ with 10Mb.

Table S6. Replication Mendelian randomization results for effect of genetically predicted fatty acid metabolites on asthma

outcome	exposure	method	P valve	beta	OR
Asthma	Arachidonate (20:4n6)	MR Egger	1.49E-04	0.87 (0.51, 1.22)	2.38 (1.66, 3.4)
		Weighted median	3.13E-05	0.65 (0.34, 0.95)	1.91 (1.41, 2.59)
		Inverse variance weighted	1.34E-05	0.52 (0.29, 0.76)	1.69 (1.33, 2.13)
Asthma	Ratio of polyunsaturated fatty acids to total fatty acids	MR Egger	3.21E-02	0.14 (0.02, 0.26)	1.15 (1.02, 1.3)
		Weighted median	9.04E-02	0.07 (-0.01, 0.15)	1.07 (0.99, 1.16)
		Inverse variance weighted	3.01E-03	0.11 (0.04, 0.18)	1.11 (1.04, 1.19)

Asthma	Ratio of docosahexaenoic acid to total fatty acids	MR Egger	6.45E-03	0.15 (0.05, 0.25)	1.16 (1.05, 1.28)
		Weighted median	3.37E-08	0.14 (0.09, 0.19)	1.15 (1.1, 1.21)
		Inverse variance weighted	1.80E-03	0.11 (0.04, 0.18)	1.12 (1.04, 1.2)
Asthma	Degree of unsaturation	MR Egger	5.06E-03	0.14 (0.05, 0.23)	1.14 (1.05, 1.25)
		Weighted median	1.26E-05	0.11 (0.06, 0.16)	1.12 (1.06, 1.18)
		Inverse variance weighted	2.29E-03	0.1 (0.03, 0.16)	1.1 (1.04, 1.17)

Table S7. cis-eQTL-MR results and heterogeneity analysis for effect of genetically predicted expression level of *FADS1* gene on asthma

**outcome**	**exposure**	**method**	**MR analysis**			**Heterogeneity test**		$P_{MR\text{-}Egger\ intercept}$	$P_{MR\text{-}PRESSO}$
			P valve	beta	OR	Cochran's Q	P		
Asthma	FADS1	MR Egger	2.94E-01	-0.04 (-0.1, 0.03)	0.96 (0.9, 1.03)	8.81	0.18	0.304	0.36
		Weighted median	1.12E-11	-0.08 (-0.1, -0.06)	0.92 (0.9, 0.94)				
		Inverse variance weighted	4.99E-09	-0.07 (-0.1, -0.05)	0.93 (0.91, 0.95)	10.67	0.15		

Note. MR, Mendelian Randomization. *FADS1, fatty acid desaturase 1*

Supplementary Figure

Figure S1. Volcano plot showing effects of fatty acid metabolites on asthma.

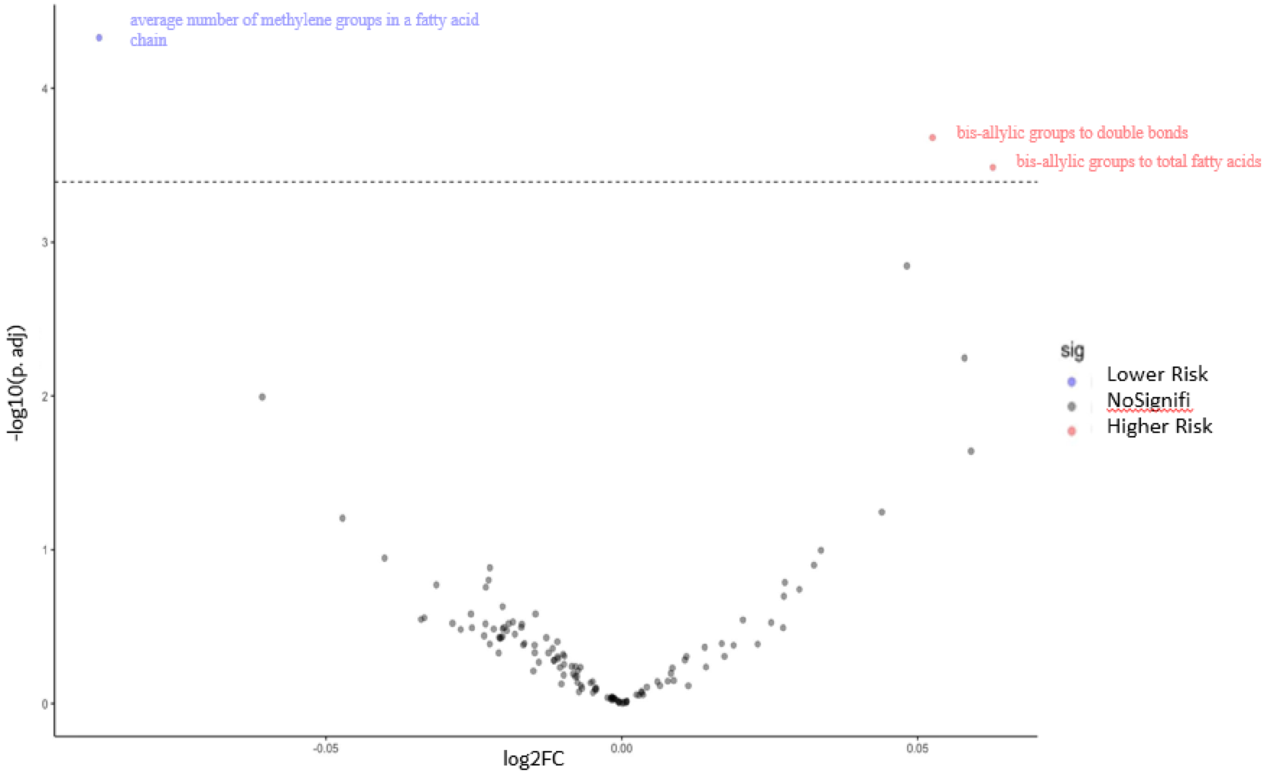

Note. Data are expressed as raw odds ratios estimated by the inverse variance-weighted (IVW) method. The black dotted line represents the association threshold of P < 0.05/123.

Figure S2. Funnel plot for the ratio of bis-allylic groups to double bonds related genetic predictors.

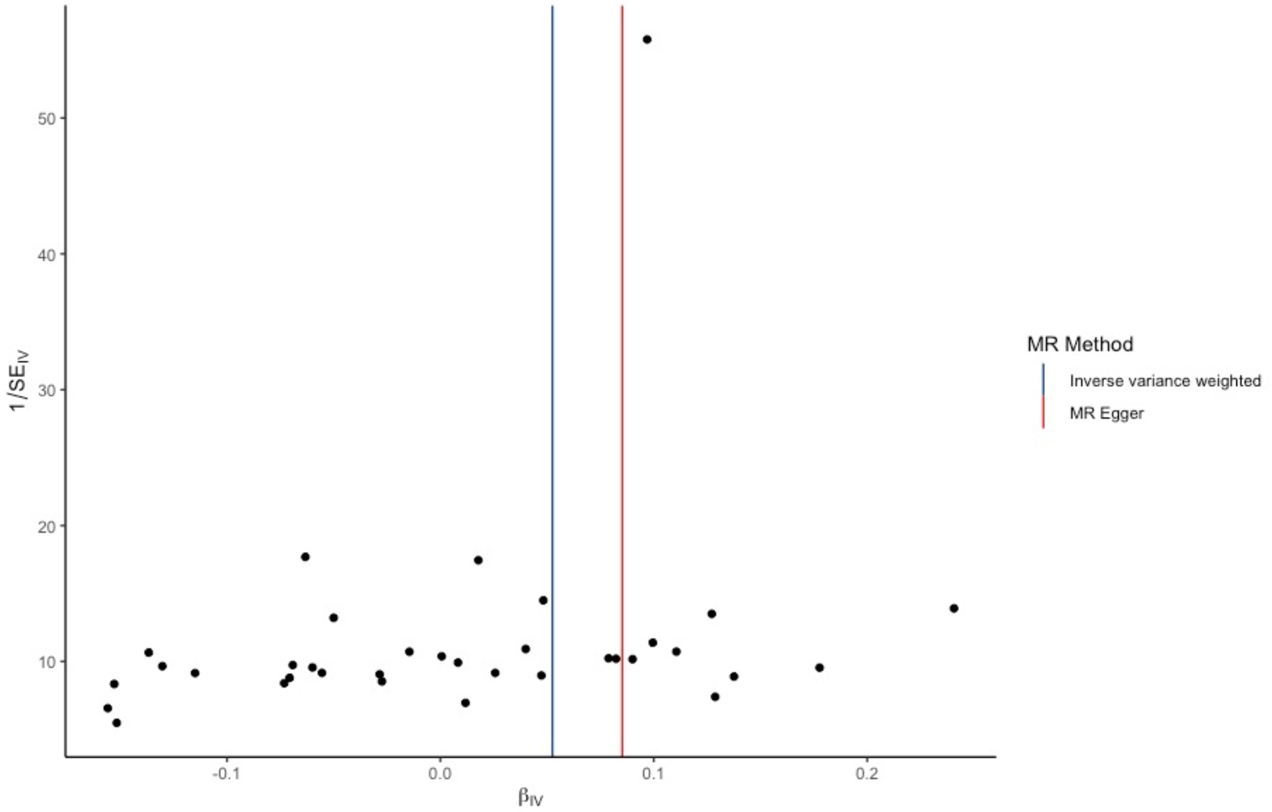

Figure S3. Funnel plot for the ratio of bis-allylic groups to total fatty acids related genetic predictors.

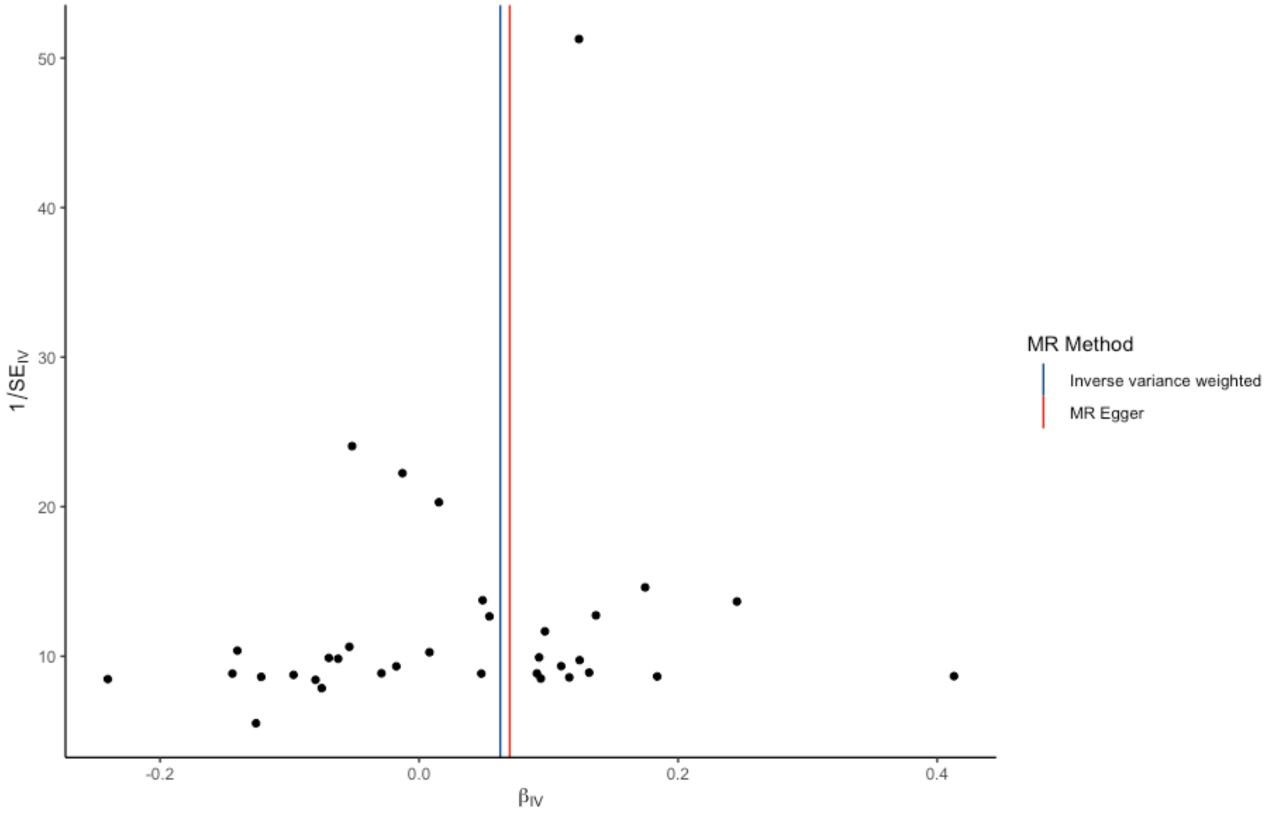

Figure S4. Funnel plot for average number of methylene groups in a fatty acid chain related genetic predictors.

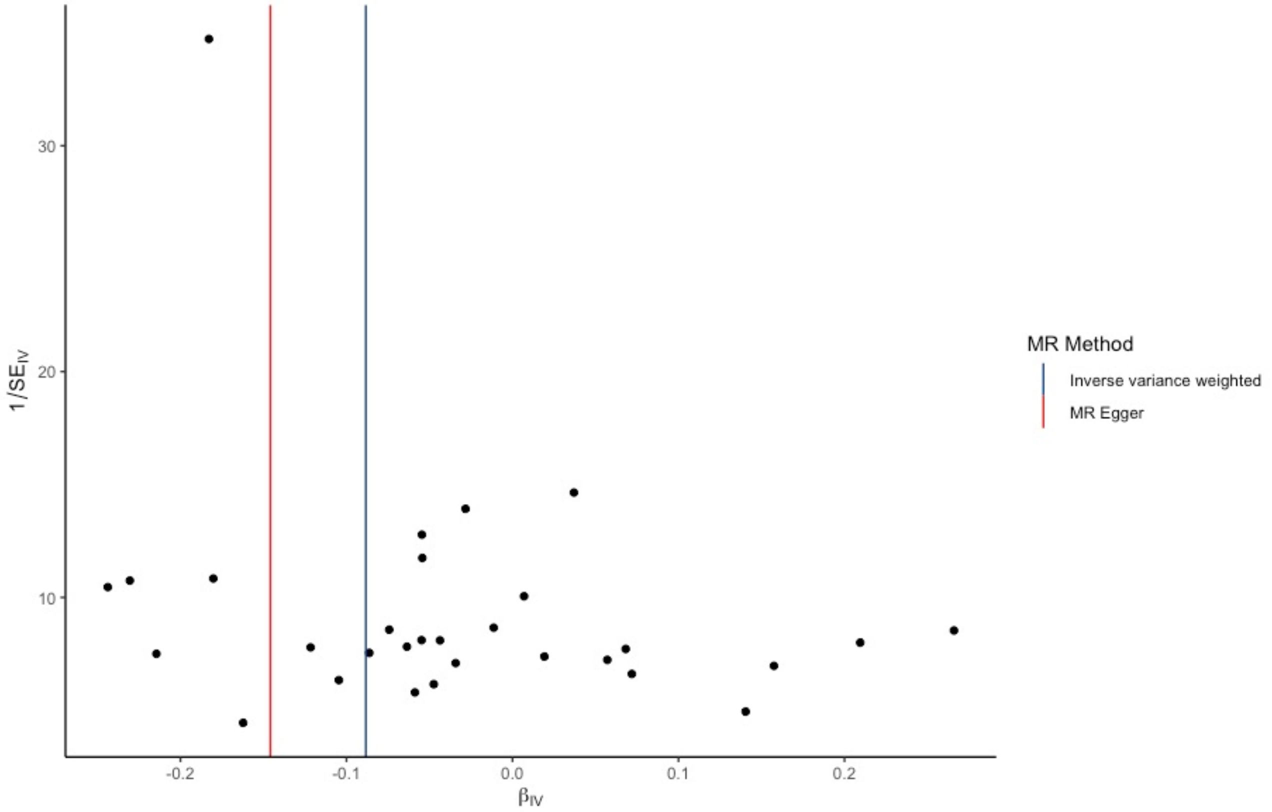

Figure S5. Replication MR analysis for the effect of metabolites on asthma using GWAS Human blood metabolites analysed by Shin et al 2014

A

Trait	Odds Ratios (OR)	P value	Q value
Oleate (18:1n9)	1.12 (0.85, 1.49)	0.41	0.88
X-12990--docosapentaenoic acid (n6-DPA)	1.02 (0.83, 1.27)	0.82	0.88
Heptanoate (7:0)	0.82 (0.63, 1.07)	0.15	0.88
Palmitate (16:0)	0.98 (0.75, 1.28)	0.88	0.9
10-heptadecenoate (17:1n7)	0.75 (0.55, 1.03)	0.07	0.77
Eicosenoate (20:1n9 or 11)	1.14 (0.9, 1.44)	0.29	0.88
Arachidonate (20:4n6)	1.69 (1.33, 2.13)	< 0.01	< 0.01
Dihomo-linolenate (20:3n3 or n6)	0.86 (0.67, 1.11)	0.24	0.88
Linoleate (18:2n6)	0.91 (0.69, 1.21)	0.54	0.88
Nonadecanoate (19:0)	1.06 (0.88, 1.29)	0.52	0.88
10-undecenoate (11:1n1)	0.98 (0.89, 1.08)	0.68	0.88
Pentadecanoate (15:0)	0.97 (0.73, 1.28)	0.81	0.88
Stearidonate (18:4n3)	1.14 (0.75, 1.72)	0.54	0.88
Adrenate (22:4n6)	1.2 (0.78, 1.85)	0.41	0.88
Margarate (17:0)	1.13 (0.85, 1.5)	0.38	0.88
Stearate (18:0)	0.87 (0.68, 1.13)	0.31	0.88
Palmitoleate (16:1n7)	1.05 (0.86, 1.29)	0.63	0.88
Myristate (14:0)	0.98 (0.8, 1.2)	0.82	0.88
Myristoleate (14:1n5)	0.83 (0.71, 0.97)	0.02	0.32
Docosahexaenoate (DHA; 22:6n3)	0.95 (0.71, 1.27)	0.74	0.88
Caprylate (8:0)	1.04 (0.85, 1.28)	0.7	0.88
Laurate (12:0)	1.12 (0.88, 1.41)	0.36	0.88
Caproate (6:0)	1 (0.73, 1.37)	0.99	0.99
Eicosapentaenoate (EPA; 20:5n3)	1.26 (0.92, 1.73)	0.15	0.88
Pelargonate (9:0)	0.9 (0.68, 1.21)	0.5	0.88
5-dodecenoate (12:1n7)	1.04 (0.91, 1.18)	0.56	0.88
10-nonadecenoate (19:1n9)	1.11 (0.82, 1.51)	0.51	0.88
Dihomo-linoleate (20:2n6)	1.03 (0.81, 1.3)	0.8	0.88
Undecanoate (11:0)	0.93 (0.7, 1.23)	0.61	0.88
Linolenate [alpha or gamma; (18:3n3 or 6)]	1.05 (0.81, 1.37)	0.69	0.88
Docosapentaenoate (n3 DPA; 22:5n3)	1.23 (0.87, 1.75)	0.24	0.88

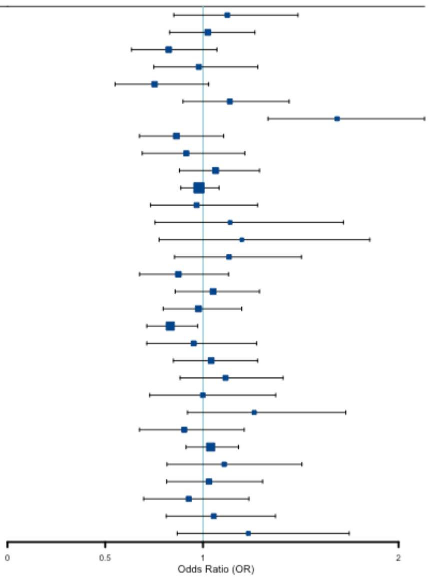

B

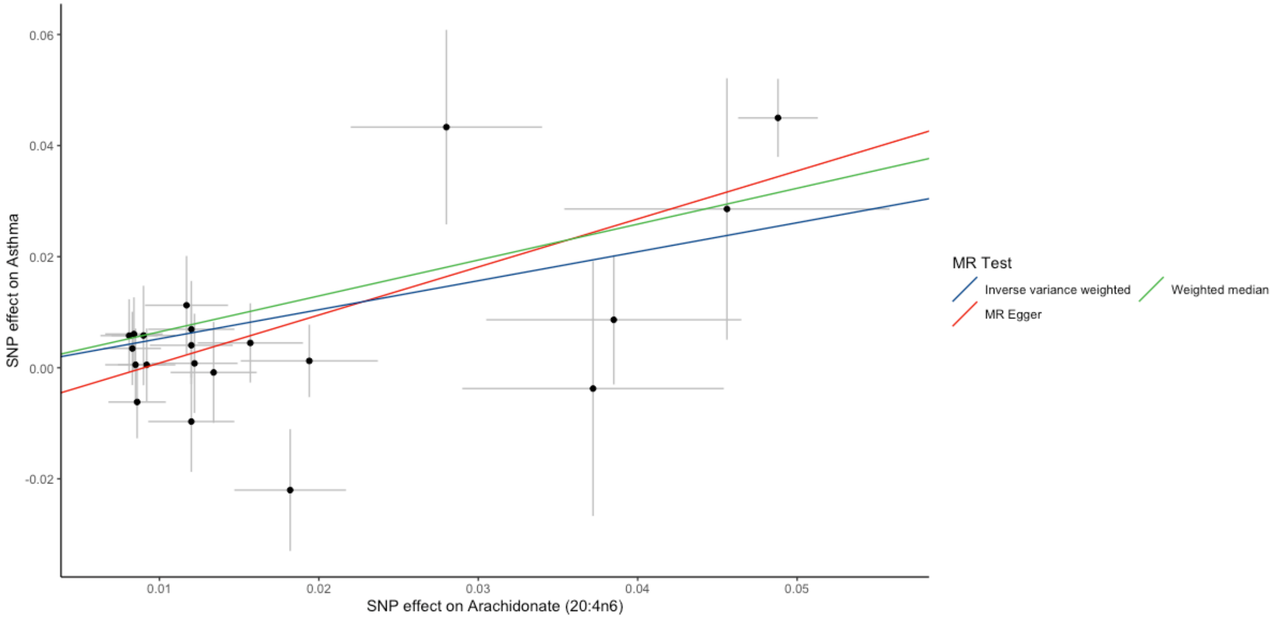
0.06
0.04
0.02
0.00
-0.02
SNP effect on Asthma
0.01
0.02
0.03
0.04
0.05
SNP effect on Arachidonate (20:4n6)
MR Test
Inverse variance weighted
MR Egger
Weighted median

C

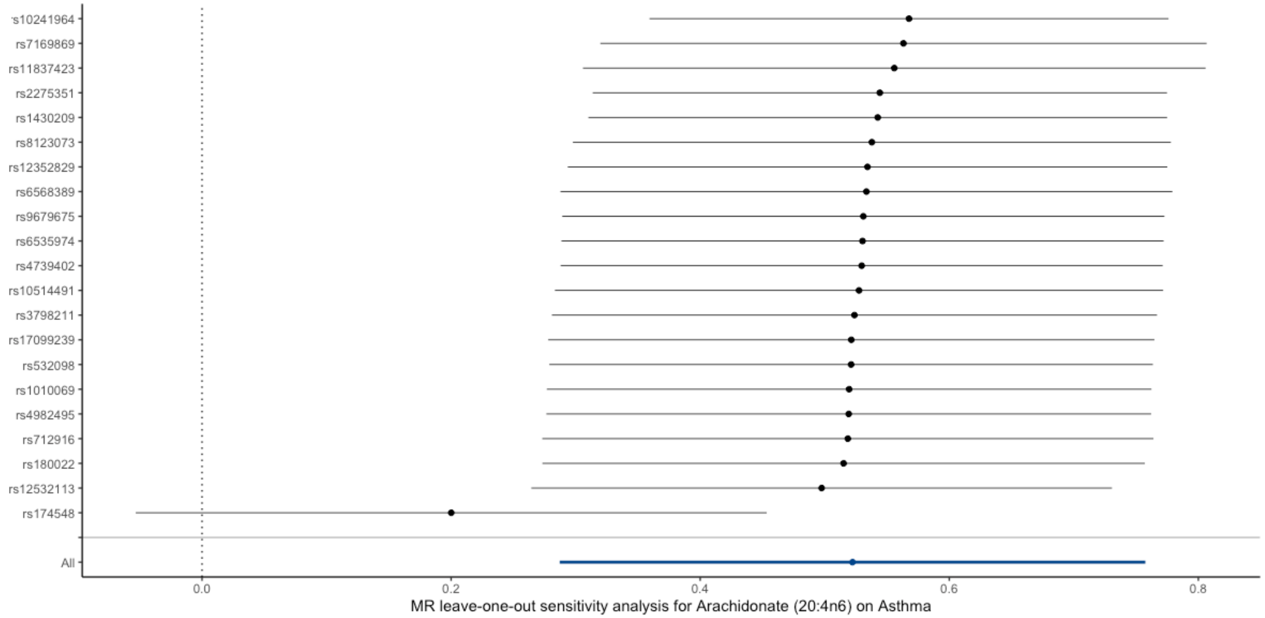

MR, mendelian randomization; OR, odds ratio; SNPs, single nucleotide polymorphisms;

SNPs with a threshold of $P < 1\times10^{-5}$ were selected as instrumental variables and used for LD clumping.

Figure S6. Replication MR analysis for the effect of metabolites on asthma using GWAS [Metabolic biomarkers in the UK Biobank measured by Nightingale Health 2020]

A

Trait	Odds Ratios (OR)	P value	Q value
Docosahexaenoic acid	1.07 (1.01, 1.14)	0.03	0.09
Ratio of docosahexaenoic acid to total fatty acids	1.12 (1.04, 1.2)	< 0.01	0.02
Linoleic acid	0.92 (0.83, 1.01)	0.09	0.15
Ratio of linoleic acid to total fatty acids	0.93 (0.87, 0.99)	0.04	0.09
Monounsaturated fatty acids	0.94 (0.88, 1)	0.06	0.11
Ratio of monounsaturated fatty acids to total fatty acids	0.94 (0.89, 0.99)	0.03	0.09
Omega-3 fatty acids	1.05 (0.98, 1.13)	0.2	0.27
Ratio of omega-3 fatty acids to total fatty acids	1.08 (1, 1.17)	0.04	0.09
Omega-6 fatty acids	0.96 (0.91, 1.02)	0.17	0.27
Ratio of omega-6 fatty acids to omega-3 fatty acids	0.94 (0.85, 1.04)	0.22	0.28
Ratio of omega-6 fatty acids to total fatty acids	1.02 (0.96, 1.09)	0.52	0.59
Polyunsaturated fatty acids	0.99 (0.93, 1.06)	0.81	0.85
Ratio of polyunsaturated fatty acids to monounsaturated fatty acids	1.08 (1.02, 1.15)	0.01	0.06
Ratio of polyunsaturated fatty acids to total fatty acids	1.11 (1.04, 1.19)	< 0.01	0.02
Saturated fatty acids	0.96 (0.89, 1.03)	0.23	0.28
Ratio of saturated fatty acids to total fatty acids	0.99 (0.87, 1.12)	0.85	0.85
Total fatty acids	0.96 (0.9, 1.02)	0.2	0.27
Degree of unsaturation	1.1 (1.04, 1.17)	< 0.01	0.02

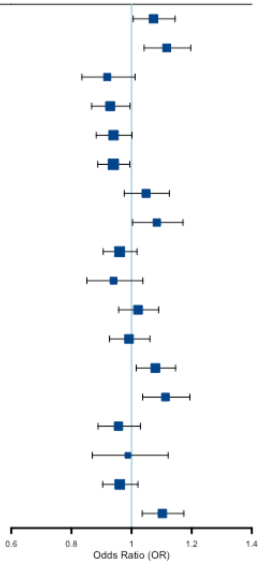

B

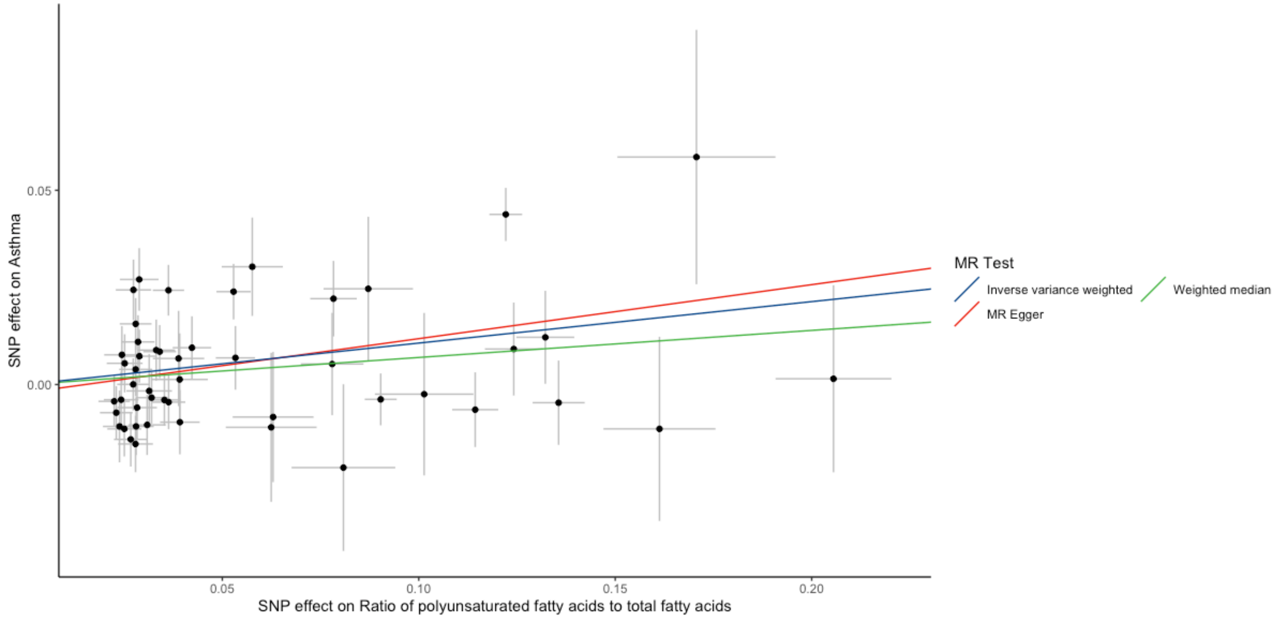
SNP effect on Asthma
0.05
0.00
0.05
0.10
0.15
0.20
SNP effect on Ratio of polyunsaturated fatty acids to total fatty acids
MR Test
Inverse variance weighted
MR Egger
Weighted median

C

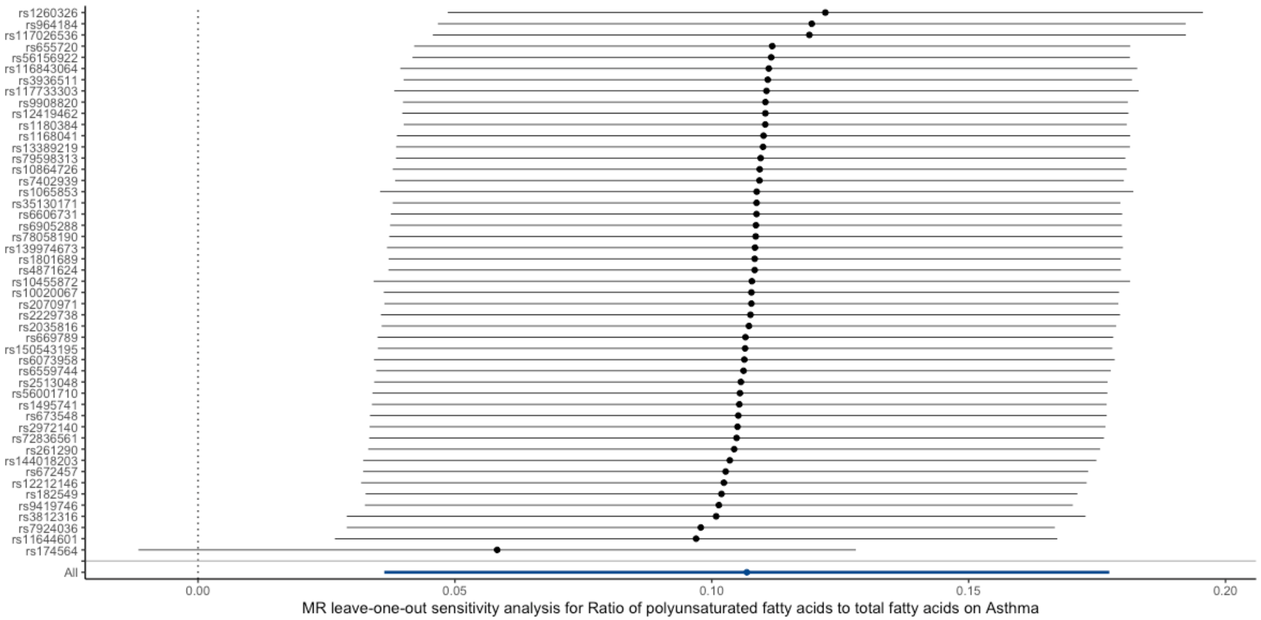
rs1260326
rs964184
rs117026536
rs655720
rs56156922
rs116843064
rs3936511
rs117733303
rs9908820
rs12419462
rs1180384
rs1168041
rs13389219
rs79598313
rs10864726
rs7402939
rs1065853
rs35130171
rs6606731
rs6905288
rs78058190
rs139974673
rs1801689
rs4871624
rs10455872
rs10020067
rs2070971
rs2229738
rs2035816
rs669789
rs150543195
rs6073958
rs6559744
rs2513048
rs56001710
rs1495741
rs673548
rs2972140
rs72836561
rs261290
rs144018203
rs672457
rs12212146
rs182549
rs9419746
rs3812316
rs7924036
rs11644601
rs174564
All
0.00
0.05
0.10
0.15
0.20
MR leave-one-out sensitivity analysis for Ratio of polyunsaturated fatty acids to total fatty acids on Asthma

D

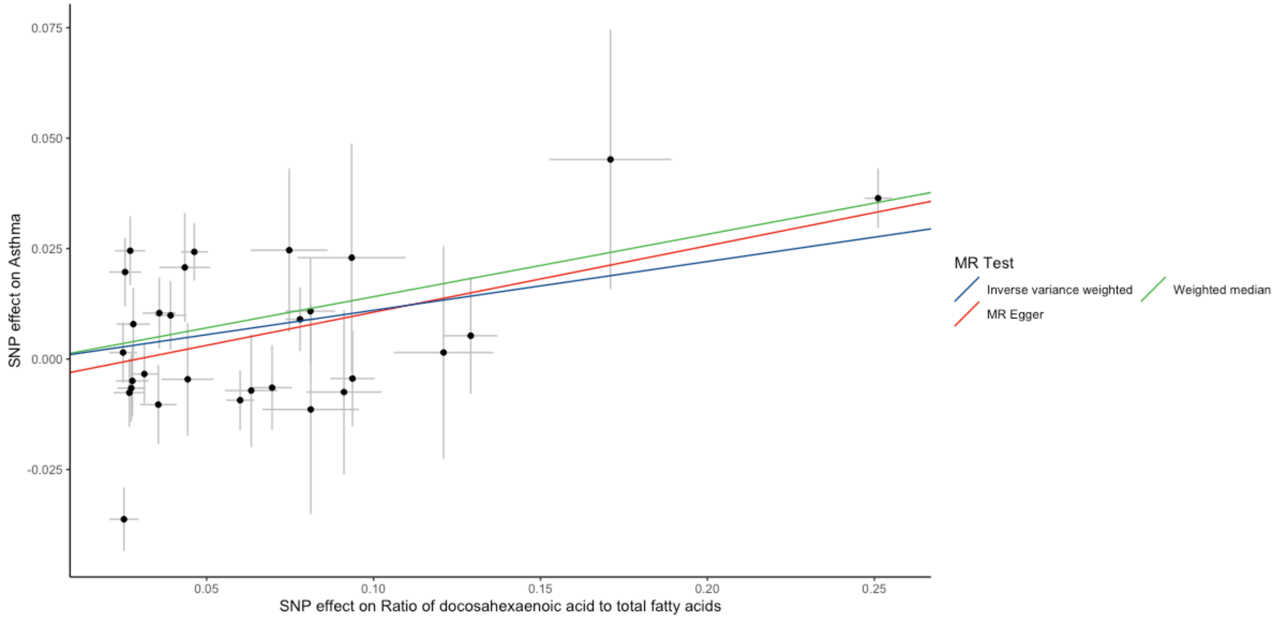

0.075
0.050
0.025
0.000
-0.025
SNP effect on Asthma
0.05
0.10
0.15
0.20
0.25
SNP effect on Ratio of docosahexaenoic acid to total fatty acids
MR Test
Inverse variance weighted
MR Egger
Weighted median

E

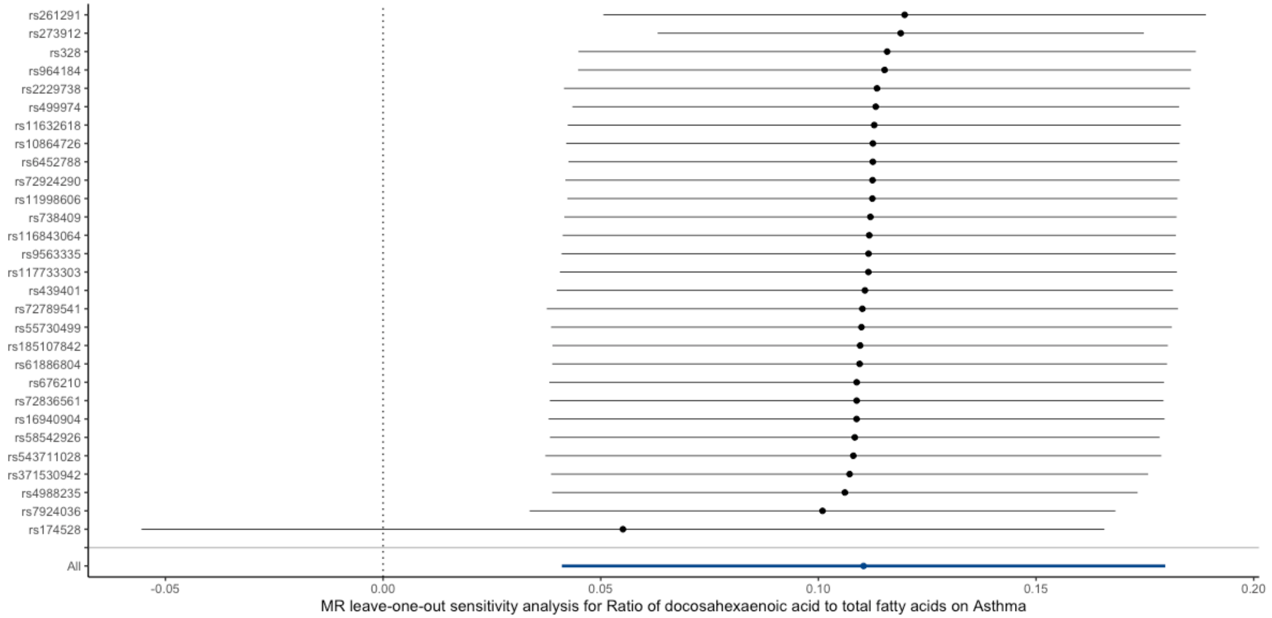

rs261291
rs273912
rs328
rs964184
rs2229738
rs499974
rs11632618
rs10864726
rs6452788
rs72924290
rs11998606
rs738409
rs116843064
rs9563335
rs117733303
rs439401
rs72789541
rs55730499
rs185107842
rs61886804
rs676210
rs72836561
rs16940904
rs58542926
rs543711028
rs371530942
rs4988235
rs7924036
rs174528
All
-0.05
0.00
0.05
0.10
0.15
0.20
MR leave-one-out sensitivity analysis for Ratio of docosahexaenoic acid to total fatty acids on Asthma

F

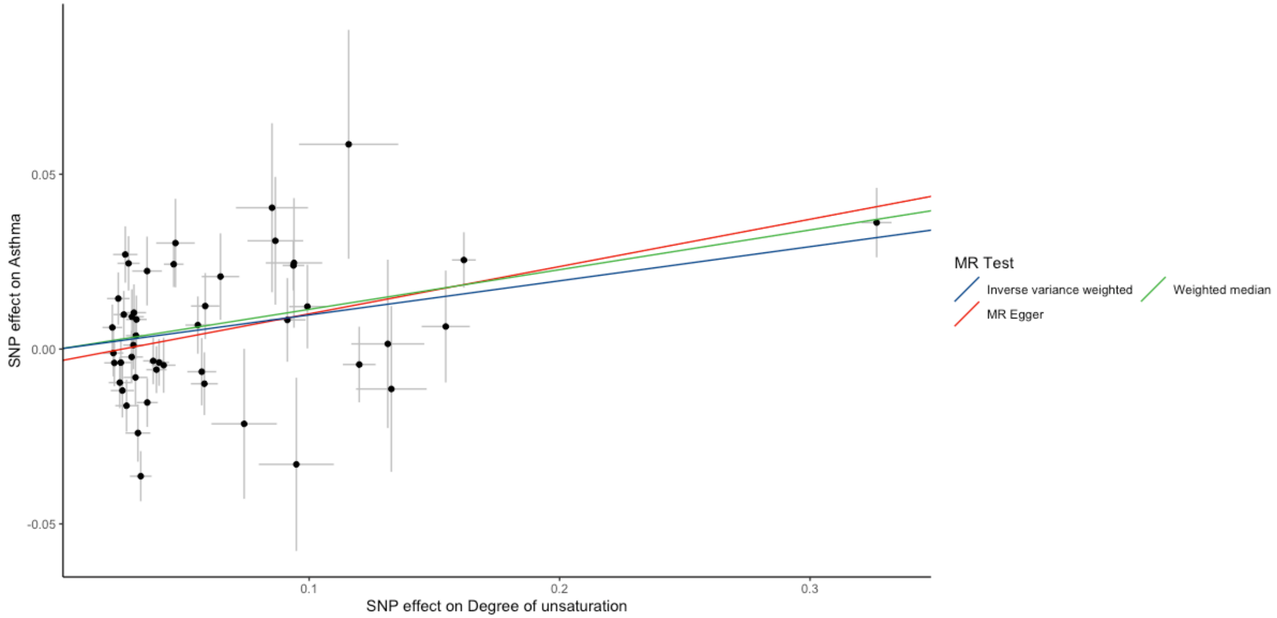

SNP effect on Asthma
0.05
0.00
-0.05
0.1
0.2
0.3
SNP effect on Degree of unsaturation
MR Test
Inverse variance weighted
MR Egger
Weighted median

G

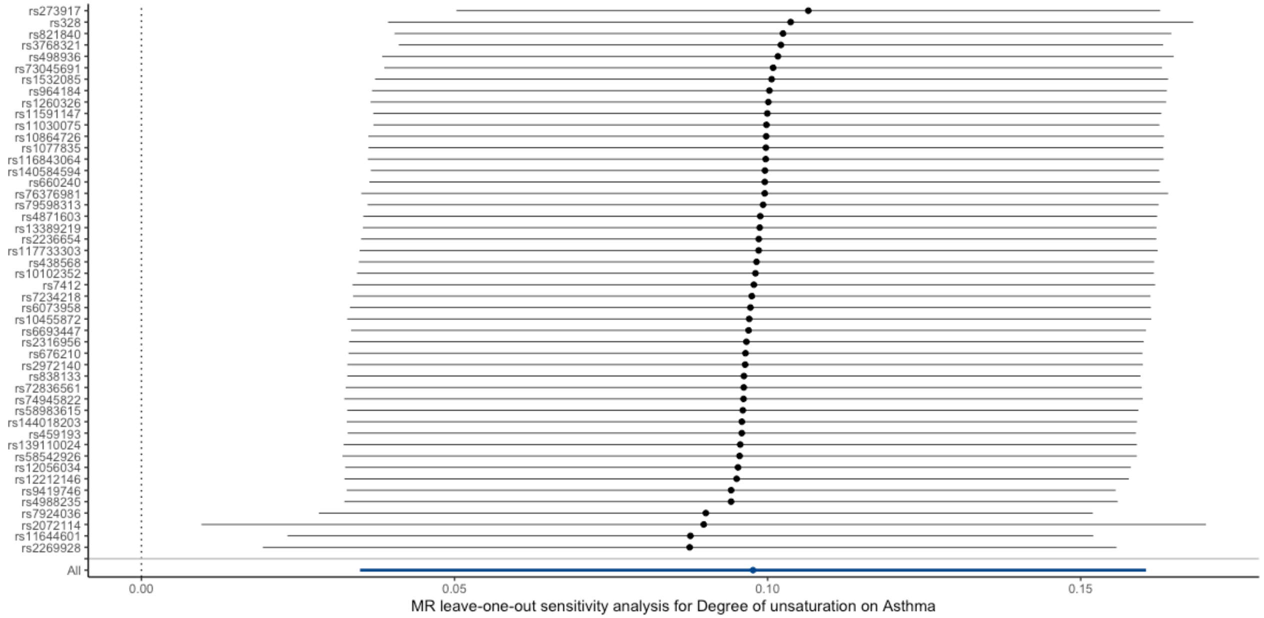

MR, mendelian randomization; OR, odds ratio; SNPs, single nucleotide polymorphisms;

SNPs with a threshold of $P < 5\times10^{-8}$ were selected as instrumental variables and used for LD clumping.

Figure S7. Scatter plot, funnel plot and forest plot of cis-eQTL-MR for the effect of FADS1 expression on asthma

A

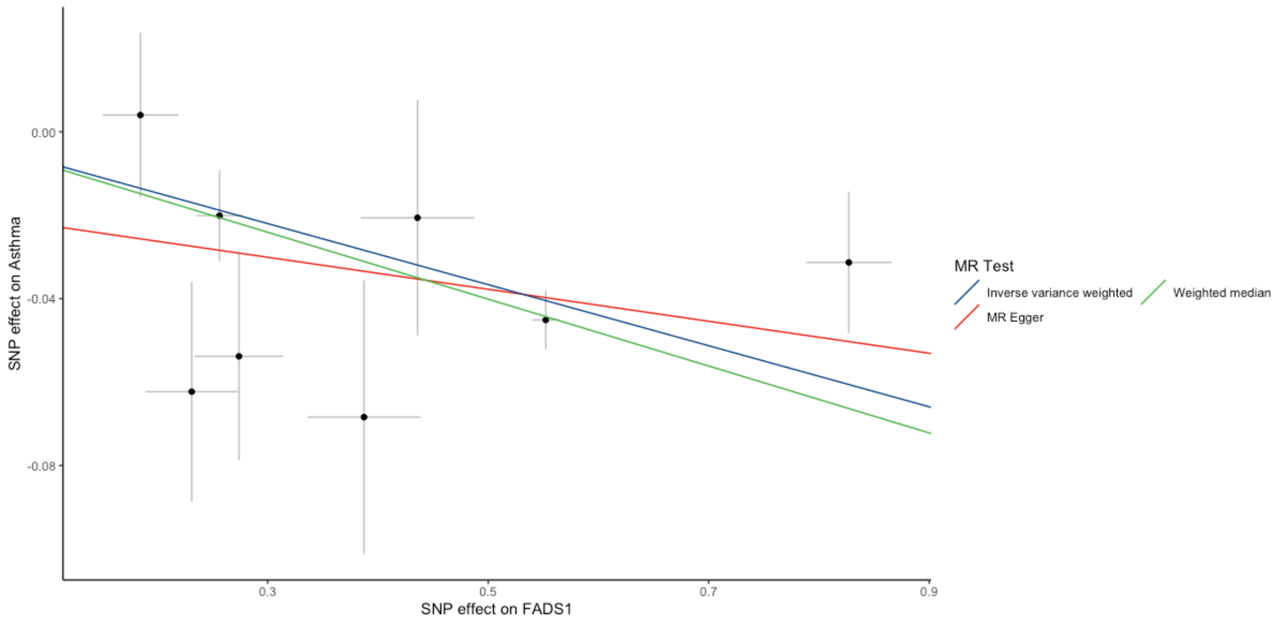

B

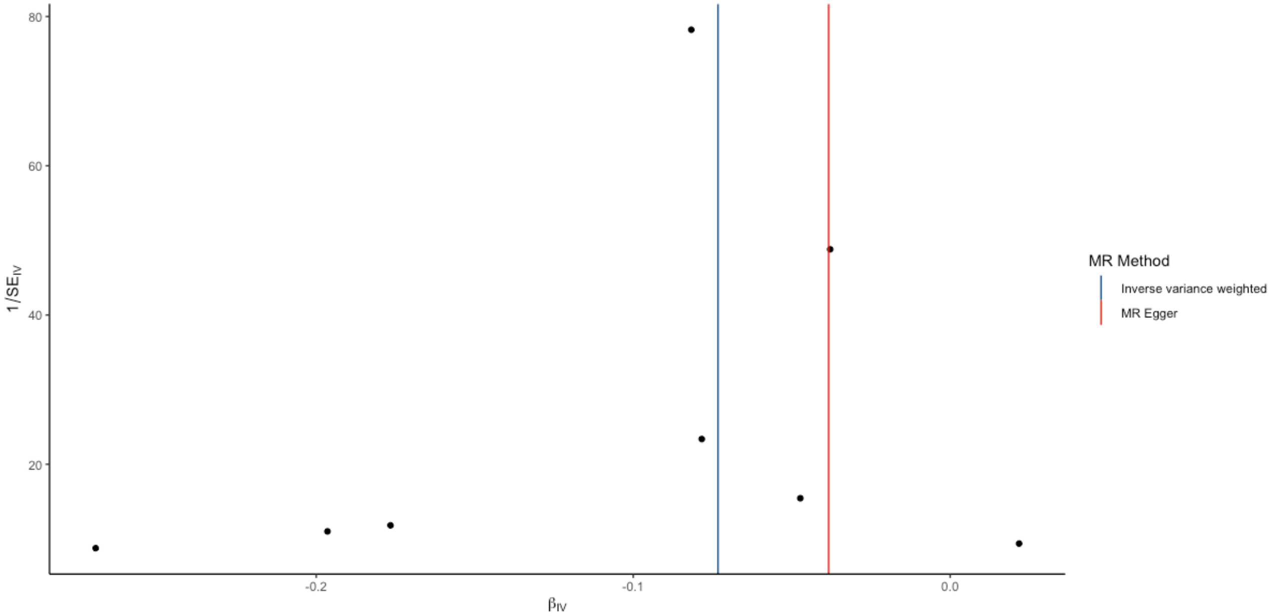

80
60
40
20
1/SE$_{IV}$
-0.2
-0.1
0.0
β$_{IV}$
MR Method
Inverse variance weighted
MR Egger

C

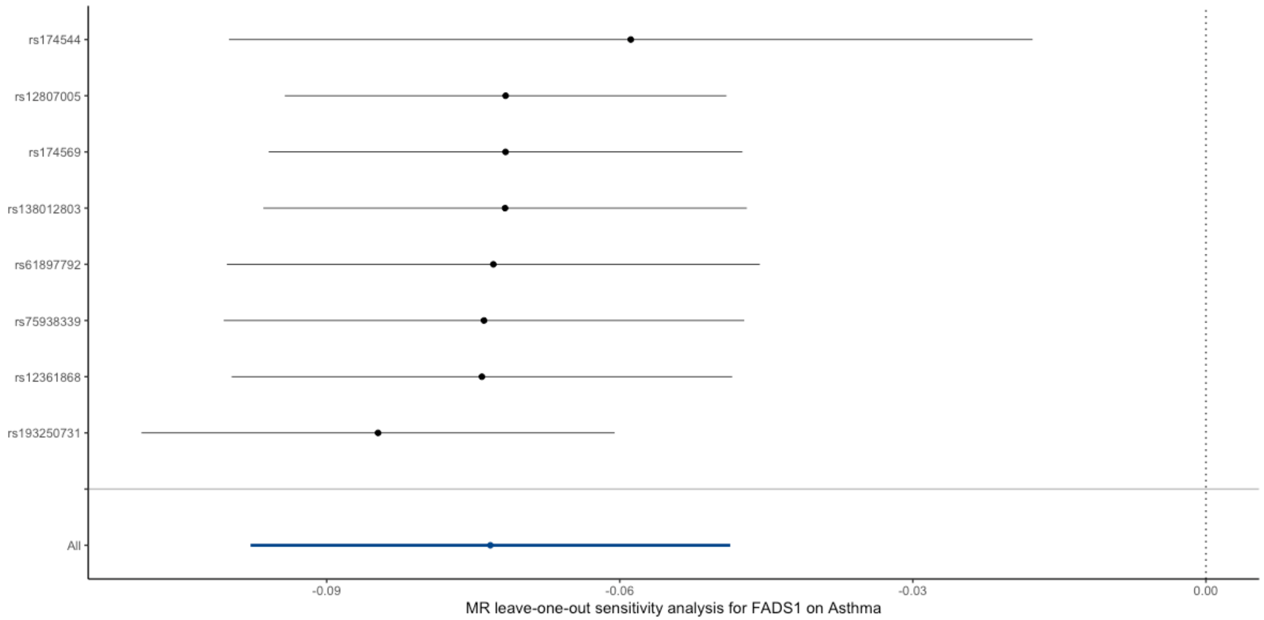

rs174544
rs12807005
rs174569
rs138012803
rs61897792
rs75938339
rs12361868
rs193250731
All
-0.09
-0.06
-0.03
0.00
MR leave-one-out sensitivity analysis for FADS1 on Asthma
